# A Computational Tool for Quantitative Analysis of Vascular Networks

**DOI:** 10.1371/journal.pone.0027385

**Published:** 2011-11-16

**Authors:** Enrique Zudaire, Laure Gambardella, Christopher Kurcz, Sonja Vermeren

**Affiliations:** 1 Angiogenesis Core Facility, Radiation Oncology Branch, National Institute of Health National Cancer Institute, Gaithersburg, Maryland, United States of America; 2 Inositide Laboratory, The Babraham Institute, Babraham Research Campus, Cambridge, United Kingdom; 3 Advanced Biomedical Computing Center - Information System Program, Science Applications International Corporation-Frederick Inc, Frederick, Maryland, United States of America; University College London, United Kingdom

## Abstract

Angiogenesis is the generation of mature vascular networks from pre-existing vessels. Angiogenesis is crucial during the organism' development, for wound healing and for the female reproductive cycle. Several murine experimental systems are well suited for studying developmental and pathological angiogenesis. They include the embryonic hindbrain, the post-natal retina and allantois explants. In these systems vascular networks are visualised by appropriate staining procedures followed by microscopical analysis. Nevertheless, quantitative assessment of angiogenesis is hampered by the lack of readily available, standardized metrics and software analysis tools. Non-automated protocols are being used widely and they are, in general, time - and labour intensive, prone to human error and do not permit computation of complex spatial metrics. We have developed a light-weight, user friendly software, AngioTool, which allows for quick, hands-off and reproducible quantification of vascular networks in microscopic images. AngioTool computes several morphological and spatial parameters including the area covered by a vascular network, the number of vessels, vessel length, vascular density and lacunarity. In addition, AngioTool calculates the so-called “branching index” (branch points / unit area), providing a measurement of the sprouting activity of a specimen of interest. We have validated AngioTool using images of embryonic murine hindbrains, post-natal retinas and allantois explants. AngioTool is open source and can be downloaded free of charge.

## Introduction

The formation of new blood vessels from a pre-existing vascular plexus is called angiogenesis. This is a complex process that depends on tight co-ordination of several important cellular activities, including proliferation, differentiation and migration [1]. In addition to being a requirement for healthy growth during development, for wound healing, the female reproductive cycle and the placenta, aberrant angiogenesis also underpins a series of pathological conditions, most notably tumour progression [2].

Recent years have seen the identification of many important regulators of angiogenesis. Due to the vital role angiogenesis plays during embryonic development, knocking out such regulators often leads to embryonic lethality, typically from mid-gestation. This restricts *in vivo* analysis of angiogenesis to earlier embryonic stages, or demands lengthy breeding of conditional knock-out systems to allow inducible deletion at later stages.

Many experimental systems allow studying different aspects of angiogenesis. These can be broadly divided into two groups. *In vitro* assays rely on cultured endothelial cells and assay a particular aspect of endothelial cell biology such as cell motility in a transwell or tube formation in a three-dimensional matrix. *In vivo* assays give a wealth of information on many aspects of endothelial cell biology and are particularly useful when genetically altered model organisms are being examined. A well-established model system in the mouse, which allows characterization of developmental sprouting angiogenesis in embryos from E10, is the embryonic hindbrain [3]. The murine post-natal retina is perhaps the most commonly used, comprehensive *in vivo* experimental system today. An advantage of retinal angiogenesis is that the effects of activators or inhibitors of proteins of interest on angiogenesis can be analysed after intravitreal or systemic administration of such substances [4]. In addition, post-natal retinal angiogenesis is commonly used for studies involving the control of tip cells, found at the leading front of new vascular sprouts, since their characteristic filopodia are particularly apparent in this system [5]. Finally, and often concurrently, the retinal model is used for the analysis of conditional knock-outs in which germ-line deletion leads to embryonic death [6]. This demands the generation of a suitable, inducible mouse model, and intravitreal or systemic administration of agents inducing deletion of the floxed gene of interest. Whilst very instructive, this is a lengthy and expensive experimental strategy due to the breeding involved.

Embryonic explants, taken at an early developmental stage (typically around E8) allow for immediate *ex vivo* analysis of developmental angiogenesis even in the majority of embryonically lethal mutants, given they are taken before the onset of embryonic wasting (typically from E9 or later). Growing in a tissue culture incubator under defined conditions, explant cultures avoid potentially deleterious external influences, such as placental defects, heart defects or hypoxia. Two types of explants are commonly used. Para-aortic splanchnopleural explants grow over a period of two weeks on a layer of OP9 feeder cells and allow distinguishing between vasculogenesis and angiogenesis defects [7]. Allantois explants grow in a fibronectin-coated tissue culture dish and produce a complex vascular network by sprouting angiogenesis in less than 24 hours [8], [9]. In common with the *in vivo* assays described above, allantois explants are useful for the analysis of several endothelial cell parameters, including cell proliferation, cell migration and sprouting.

Quantitative analysis of the vascular networks in the above systems is not standardized and tends to be done in a non-automated fashion, making the analysis process labour intensive and prone to human error. There is a lack of easily accessible and user-friendly software tools designed to perform comprehensive quantitative analysis of vascular networks. To remedy this, we designed AngioTool, a software for the quantitative analysis of angiogenesis with user-friendly interface and analysis flow. AngioTool computes several morphological and spatial parameters including the overall size of the vascular network, total and average vessel length, vascular density as well as lacunarity, which characterizes vessel non-uniformity by assessing the variation in foreground and background pixel mass densities across an image. Lacunarity is able to characterise oddities found when vessel organisation has been disturbed significantly, and may be useful to characterise and quantitatively analyze vascular networks in drug treated specimens or pathological vasculature as has been shown in patients'lung tumours where this parameter correlates with stages of aggressiveness [10]. Finally, AngioTool provides a measure of angiogenic sprouting activity by computing the “branching index” (branch points / unit area) of the vascular networks analysed. The present work describes and validates the use of AngioTool in the analysis of angiogenesis in murine embryonic hindbrains, post-natal retinas and allantois explants.

## Results

We previously analysed the function of ARAP3, a PI3K regulated dual GTPase activating protein, in angiogenesis [11]. For the analysis of vascular networks in allantois explants derived from control and *Arap3* mutant embryos, we devised a computational analysis method, which evaluated the “branching index”, the number of vessel branch points per unit area. We have since developed this into a standalone application named AngioTool, which allows performing comprehensive quantitative assessments of angiogenesis in a visually user-friendly fashion. AngioTool computes the branching index and several other morphometric parameters such as total explant area, average and total vessel length, number of endpoints, vessel density, and average lacunarity.

### AngioTool

**Figure 1:**
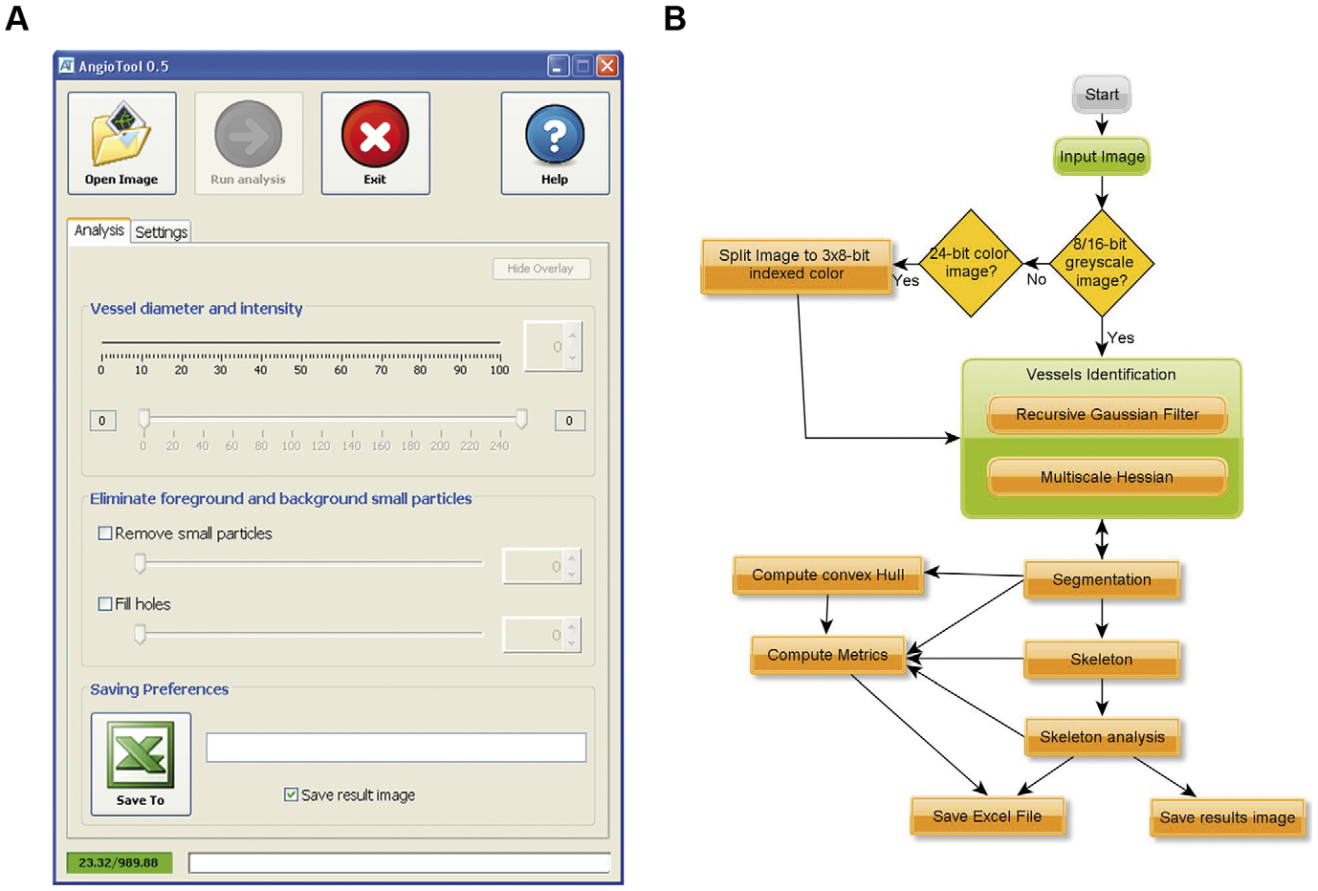
AngioTool's GUI and analysis flow. (A) AngioTool GUI for analysis of angiogenesis networks. A detailed quick analysis guide can be accessed through the help button. (B) Representation of the logical analysis flow performed by AngioTool which is based on identification of vessels using multiscale Hessian analysis and skeletonization. The analysis process is mostly automated (yellow boxes) with minimal user intervention required to select the test image and during the visual identification of vessels (green squares).

AngioTool's graphical user interface (GUI) contains a top row with buttons and two tabs containing controls to run analysis and to customize the numeric and graphical output (Fig. 1A). The basic analysis flow implemented by AngioTool includes segmentation, skeletonization and analysis of the vasculature (Fig. 1B). On opening an image, AngioTool identifies vessel profiles according to the software's preset parameters. Identified vessels are demarcated with an outline on the displayed image which dynamically updates its shape in response to adjustments done using the controls included in the analysis tab. Once the outline overlay matches the vessels in the displayed image, the analysis can be carried out. On completion of the analysis, the resulting image shows an overlay, which indicates the area encompassing all vessels, a skeletal representation of the vascular network and the computed branching points inside this area. This image is saved together with an Excel file containing the analysis parameters and the computed results. AngioTool features several controls to customize the analysis and the output image under the settings tab and a Help button that displays a guide to installation of the software and analysis steps.

### Validating AngioTool

We tested AngioTool using three different sets of images: murine embryonic hindbrains, post-natal retinas and allantois explants. All of these images are available as supplemental files.

**Figure 2:**
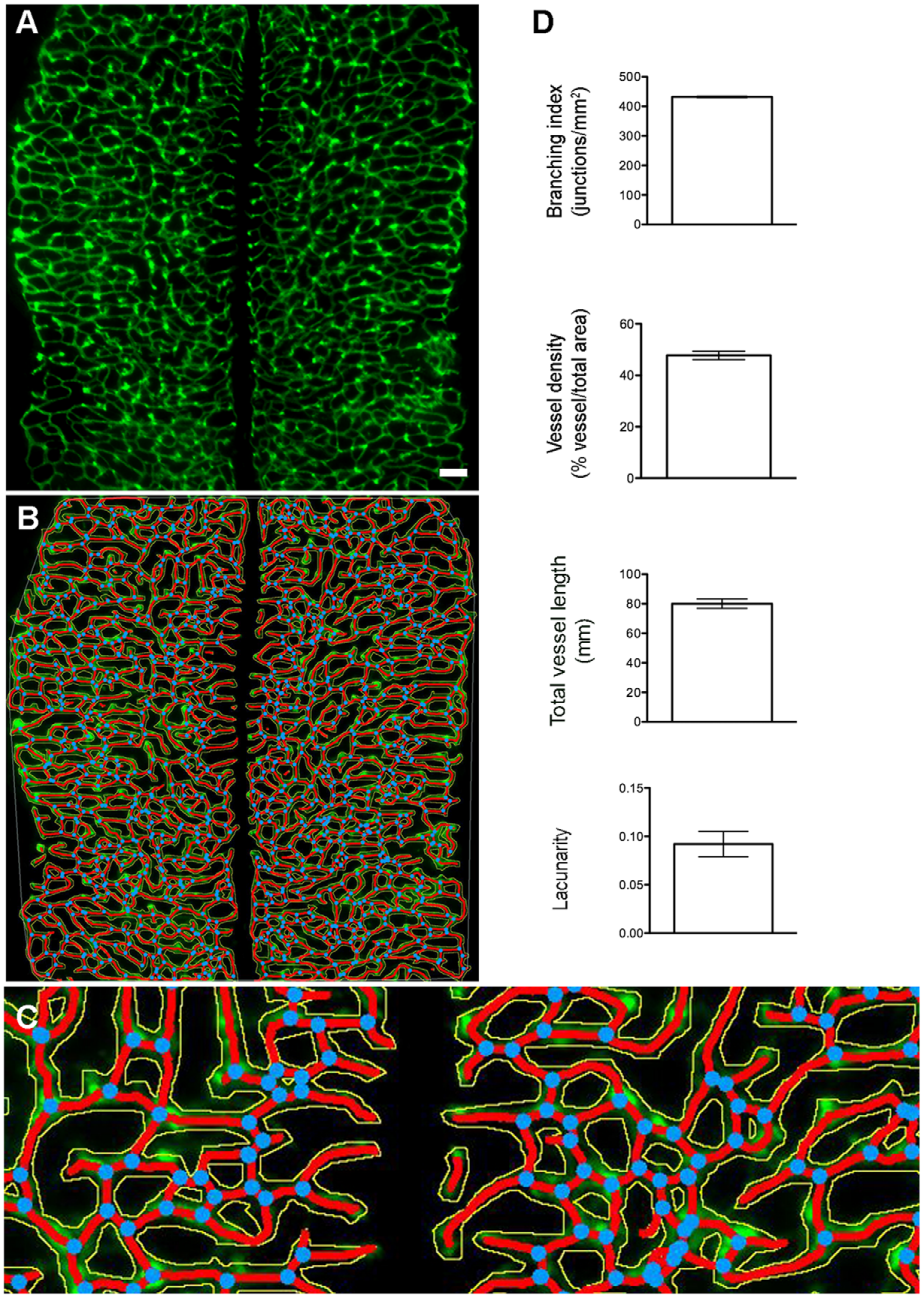
Angiogenesis in the embryonic hindbrain. Hindbrains were dissected, stained and microscopic photographs were taken and assembled as described. (A) Representative E11 hindbrain. (B) Result image after analysis of the hindbrain shown in (A). (C) Enlarged area from skeleton shown in (B). The outline of the vasculature is shown in yellow, the skeleton representation of vasculature in red and branching points are blue. (D) Graphical representation of the analysis performed on three individual hindbrains.

Endomucin-stained E11.5 embryonic hindbrains (for raw images, see Figs S1, S2, S3) show the developing subventricular plexus (Fig. 2). Generation of skeletons for these complex vascular networks required only minor adjustments to AngioTool's pre-computed parameters (Fig. 2B,C) and analysis was typically achieved within less than five minutes per image. Analysis of three separate E11.5 wild-type hindbrains (43–5 somites) with AngioTool rendered consistent results across all analysed parameters (Fig. 2D).

**Figure 3:**
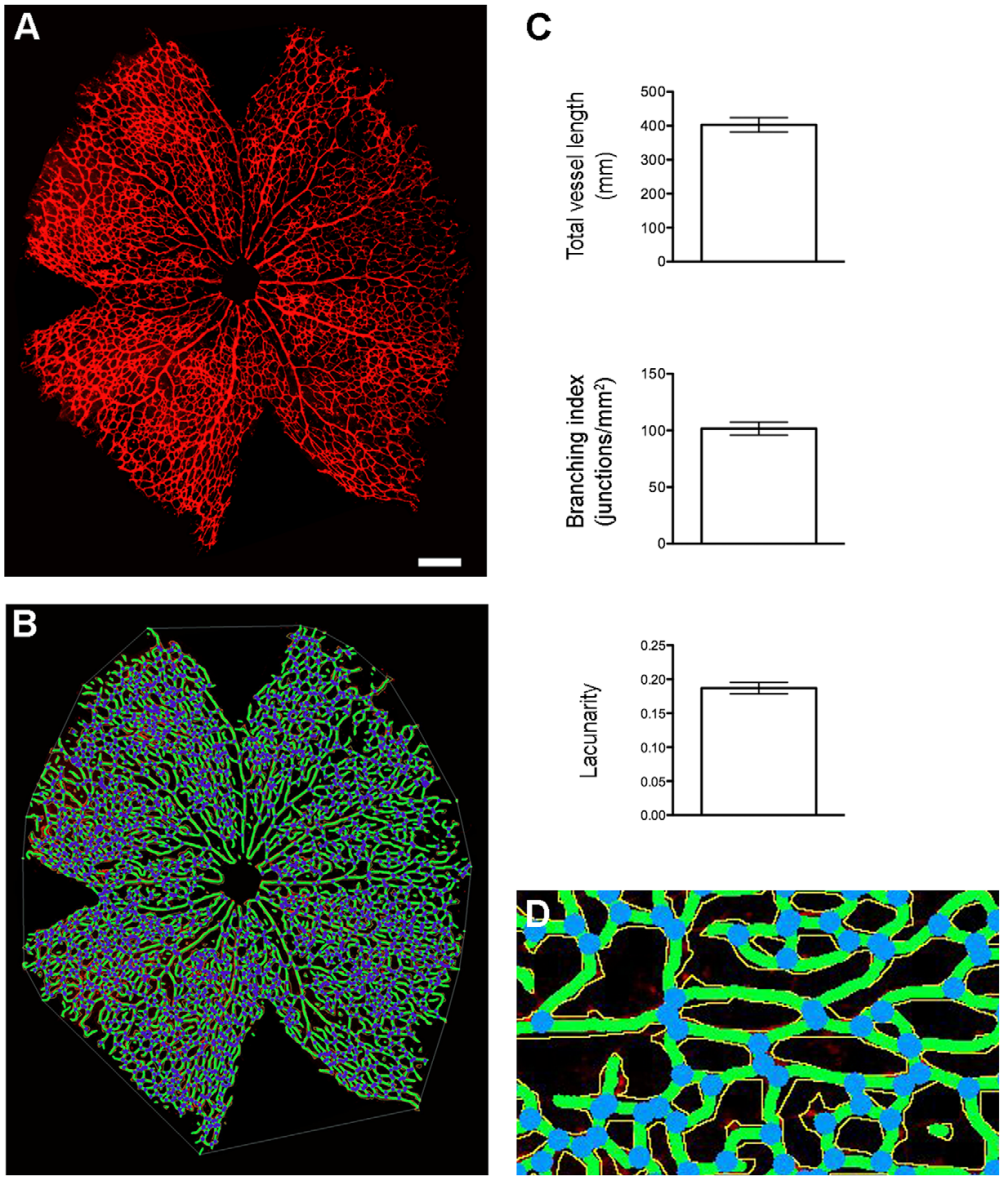
Post-natal angiogenesis in the murine retina. Retinas were dissected, stained, flat mounted and microscopic photographs were taken and assembled as described. (A) Representative P6 retina and (B) the resulting image after analysis (C) Graphical representation of the analysis performed on four individual retinas. (D) Enlarged area from (B). Vessels outlines are shown in yellow, the skeleton in green and branching points in blue.

We next stained the retinal vasculature of P6 wild-type pups using isolectin B4, visualising a large and complex vascular structure, manual analysis of which would be extremely lengthy (Fig. 3; raw images Figs S4, S5, S6, S7). As with the hindbrains images, only minor adjustments to the pre-set parameters were required and the analysis with AngioTool was typically done within less than five minutes (Fig. 3B,D). As illustrated in the graphical representations (Fig. 3C), the analysis of four P6 retinas taken from littermates produced consistent read-outs (total vessel length, branching index and lacunarity), in line with the notion that post-natal angiogenesis in these retinas should proceed at a very similar rate.

Development of vascular networks generated *ex vivo* in allantois explants follows a less predictable pattern than those found in the well-characterised developing embryonic hindbrains or post-natal retinas. Allantois explants are not only useful for the analysis of angiogenesis in mice with embryonic lethal phenotype but also amenable to the presence of agents such as inhibitors [11], [12], blocking antibodies [9], [13] or viral transduction [14] without the need for any invasive animal experimentation.

**Figure 4:**
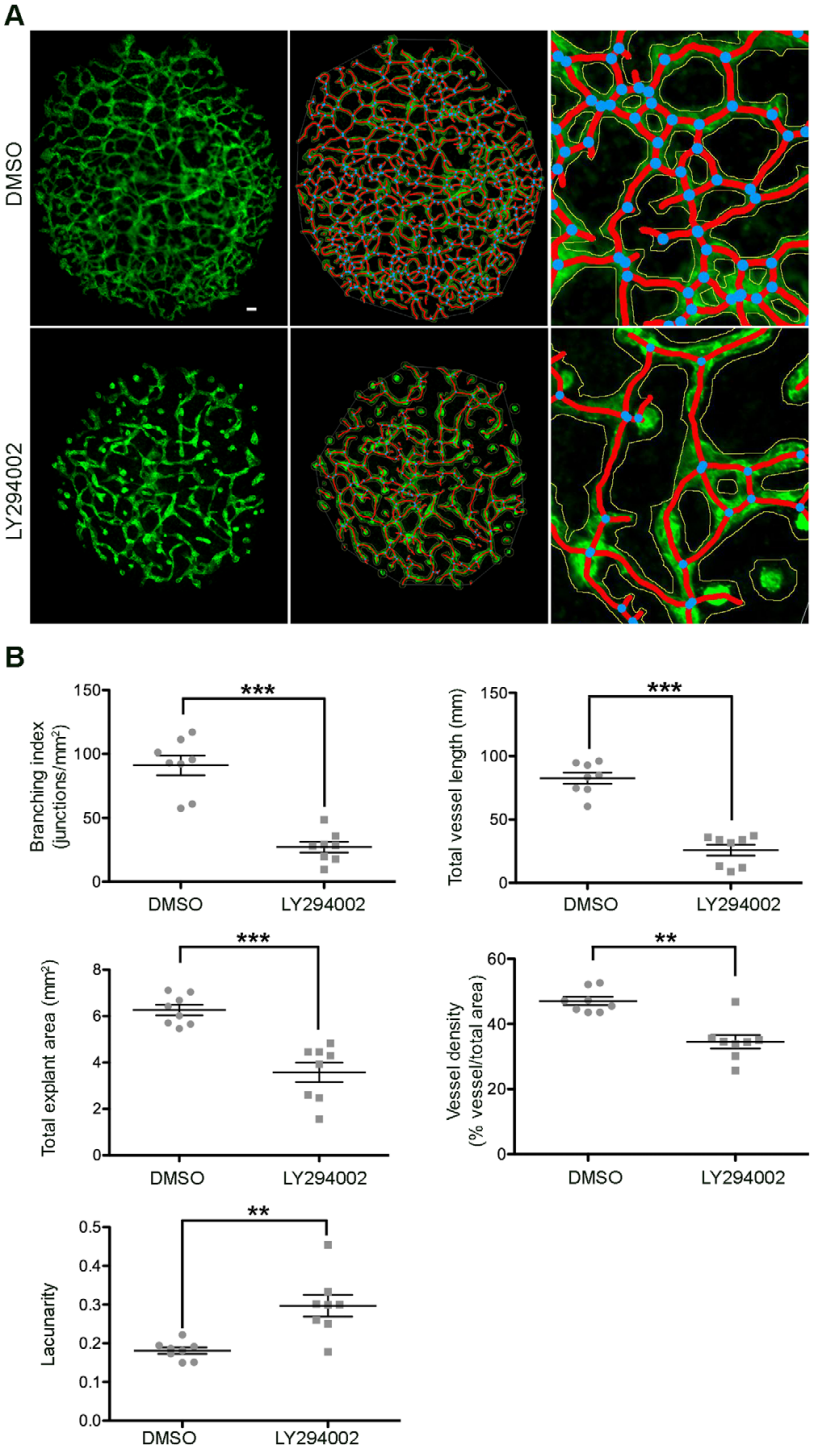
Analysis of angiogenesis in LY294002 treated allantois explants. Allantois explants were cultured in the presence of inhibitor or its vehicle, fixed, stained and microscopic photographs taken as described. (A) Representative images of a control (top) and a LY294002-treated (bottom) allantois explant (left), the resulting images after analysis (middle panels) and a representative, enlarged part of the result images shown in the middle panels. The vessel outlines are shown in yellow, the skeletons in red and the branching points in blue (B) Graphical representations of the analysis with AngioTool performed on eight control and eight LY294002-treated explants. Statistical analysis was by T-test (Mann Whitney) ***, p<0.001, **, p<0.01.

**Figure 5:**
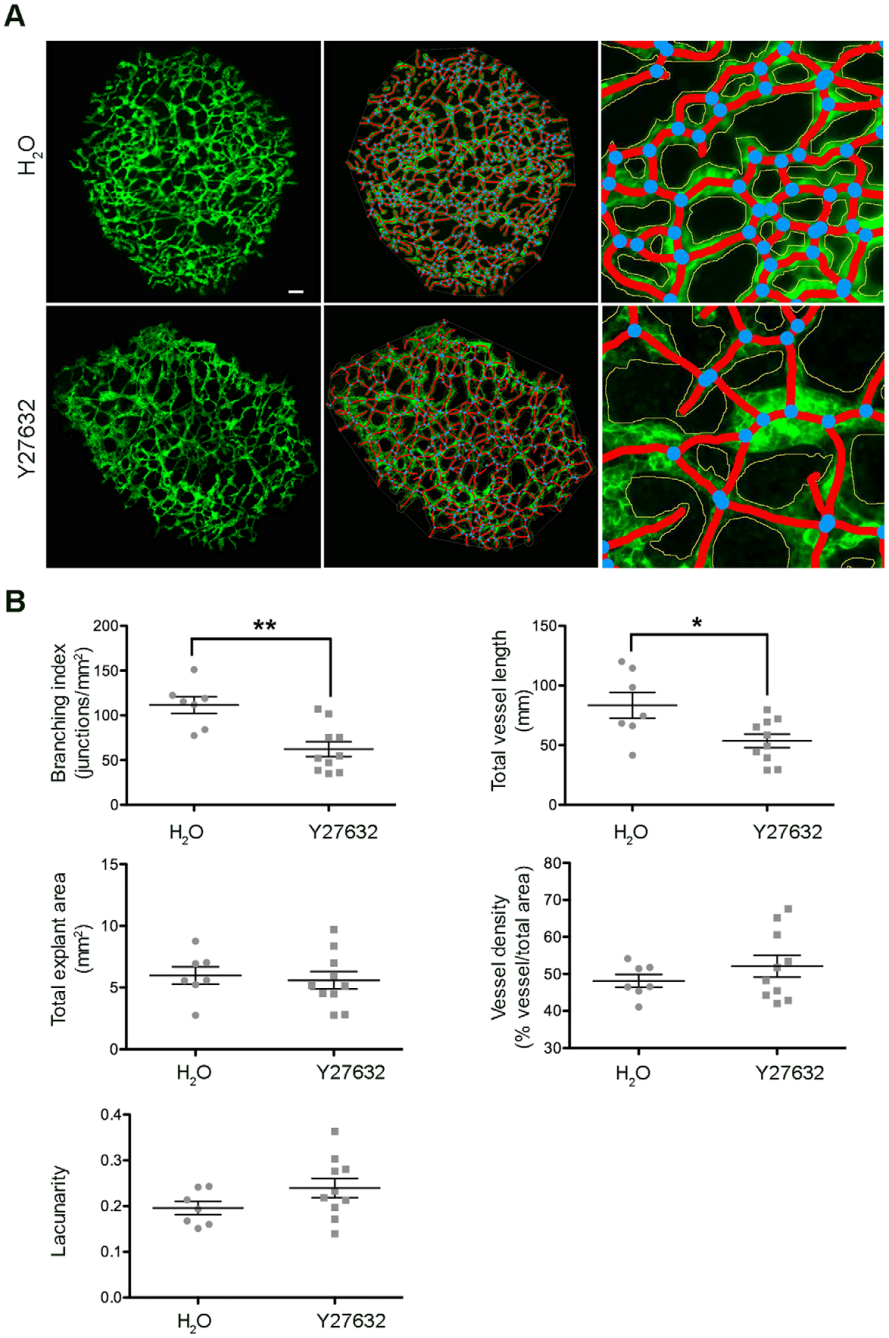
Analysis of angiogenesis in Y-27632 treated allantois explants. Allantois explants were cultured in the presence of inhibitor or its vehicle, fixed, stained and microscopic photographs taken as described. (A) Representative images of a control (top) and a Y-27632 treated (bottom) allantois explant (left), their skeletonised images (middle panels) and a representative, enlarged part of the skeletonised images shown in the middle panels. The vessels outlines are represented in yellow, the skeleton in red and the branching points in blue. (B) Graphical representations of the analysis with AngioTool performed on seven control and ten Y-27632 treated explants. Statistical analysis was by T-test (Mann Whitney) *, p<0.05; **, p<0.01.

We performed two sets of experiments, in each of which allantois explants were either treated with a well-characterised inhibitor, or with its vehicle (Figs 4, 5). Vehicle-treated allantois explants were disk-like structures that typically exhibited few large, central vessels, a large number of intermediate vessels and many small sprouts (Fig. 4A, upper panels). The first inhibitor we used was the well characterised, stable pan PI3K inhibitor LY294002 [15]. The p110alpha catalytic subunit of agonist activated PI3K has been shown to be required for both developmental and pathological angiogenesis [16], [17]. LY294002 caused a severe defect in allantois explant angiogenesis, with treated explants visually exhibiting smaller size, reduced complexity and fewer sprouts (Fig. 4A, lower panels; raw control images Figs S8, S9, S10, S11, S12, S13, S14, S15, LY294002 treated Figs S16, S17, S18, S19, S20, S21, S22, S23). Given that LY294002 is a pan-PI3K inhibitor with known off-target effects on several protein kinases [18], this striking defect was in keeping with the milder defect observed with the more p110alpha PI3K isoform selective inhibitor PI-103 used in our previous work [11].

Optimising parameters for producing skeletons that truthfully represented the structures in allantois explants was more challenging than it had been for the more evenly sized vessels in hindbrains and retinas. Accurate vessel segmentation was achieved by choosing several vessel diameter scales in combination with intensity settings that detected weakly stained structures. Occasionally, this caused segmentation of false positive structures, which were subsequently removed by elimination of small particles and careful optimization of the fill holes function such that only true vessels were labelled. In our hands, optimising explant skeletons took on average 10–20 minutes, depending on the complexity of the explant.

The analysis of eight vehicle and eight LY294002-treated allantois explants showed statistically very significant defects in all parameters analysed in the explants treated with the PI3K inhibitor: Total explant area, percentage of the area covered by vessels, total vessel length, branching index and lacunarity are all plotted in Fig. 4B. Given the severe defects of the LY294002-treated explants, this was an expected and reassuring result.

Inhibition of RhoA or of its effector ROCK have been shown to reduce vessel formation *in vitro* and *in vivo* [19]. ROCK activation was shown to lead to increased tumour invasiveness and higher blood vessel densities in an *in vivo* model [20]. Other reports indicated a pro-angiogenic effect upon ROCK blockage, with increased vessel length and lumens measured in tumours treated with ROCK inhibitor [21] and increased VEGF-induced sprouting in HUVEC spheroids [22], suggesting that ROCK may have a context-dependent role. To test the effect of ROCK inhibition on allantois explants, we used the well-characterised ROCK inhibitor Y27632 [23], [24]. In our experiment, explants treated with this inhibitor were large, though less uniformly shaped than vehicle-treated controls (Fig. 5A; raw control images Figs S24, S25, S26, S27, S28, S29, S30, Y27632 treated S31, S32, S33, S34, S35, S36, S37, S38, S39, S40). Y27632-treated explants contained areas with characteristically enlarged vessels, often juxtaposed to very fine vessels; the extent of these features was variable between individual Y27632-treated explants. AngioTool allowed for careful adjustment of parameters in order to efficiently detect fine vessels, whilst avoiding artifacts in distended areas on careful selection of vessel diameter, intensity and particle criteria (Fig. 4A, enlarged right hand panels). The analysis performed by AngioTool confirmed that treatment with Y27632 caused explants to have a significantly reduced branching index, and reduced total vessel length (Fig. 5B). As expected, the analysis showed no differences in the total explant area. Similarly, trends of increased lacunarity and vessel density in ROCK inhibitor treated explants were not statistically significant. Whilst we are not aware of experiments performed with ROCK inhibitor treated allantois explants elsewhere, our results support a subtle, and likely context-dependent effect of ROCK inhibition on sprouting angiogenesis, in-line with the results reported by others in a range of different assay systems [19], [20], [21], [22].

### Strengths and Weaknesses

Despite being originally devised for analysis of allantois explants, the usefulness of AngioTool extends to other assay systems, such as those found in the murine embryonic hindbrain or post-natal retinas. As with any automated assessment, AngioTool reduces subjectivity and likelihood of human error and streamlines analysis of features which could alternatively be performed manually, such as counting the number of endpoints or numbers of junctions per image. Other features of the analyses performed would be very hard to perform manually (vessel density, sprouting index) since they are normalised to the area of the analysed network. To our minds, these features make AngioTool particularly useful.

**Figure 6:**
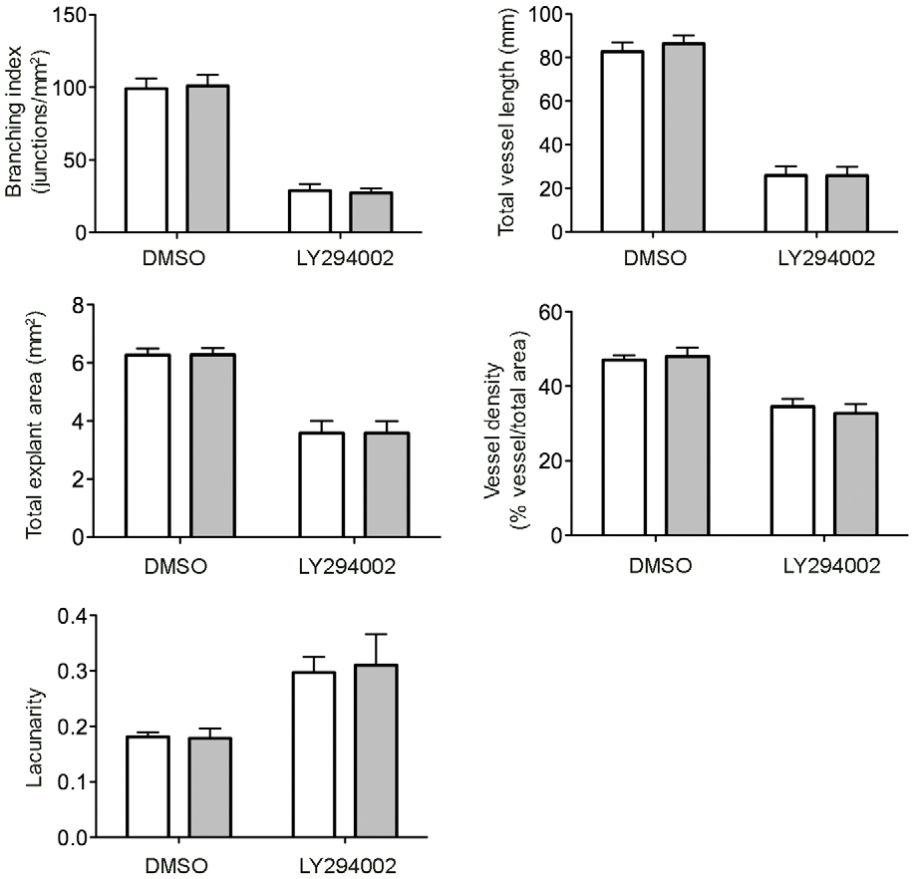
Reproducibility of image analysis using AngioTool. The eight images of control DMSO- and LY294002-treated allantois explants (see Fig. 4) were analysed independently by two investigators. The results obtained are plotted. White bars, analysis performed by person 1, grey bars, analysis performed by person 2. Results obtained were subjected to statistical analysis by 2-way ANOVA. This showed highly significant differences for LY294002 treatment under all conditions tested (p<0.001) whilst there were no significant differences between the results obtained by the two investigators (p>0.66).

**Figure 7:**
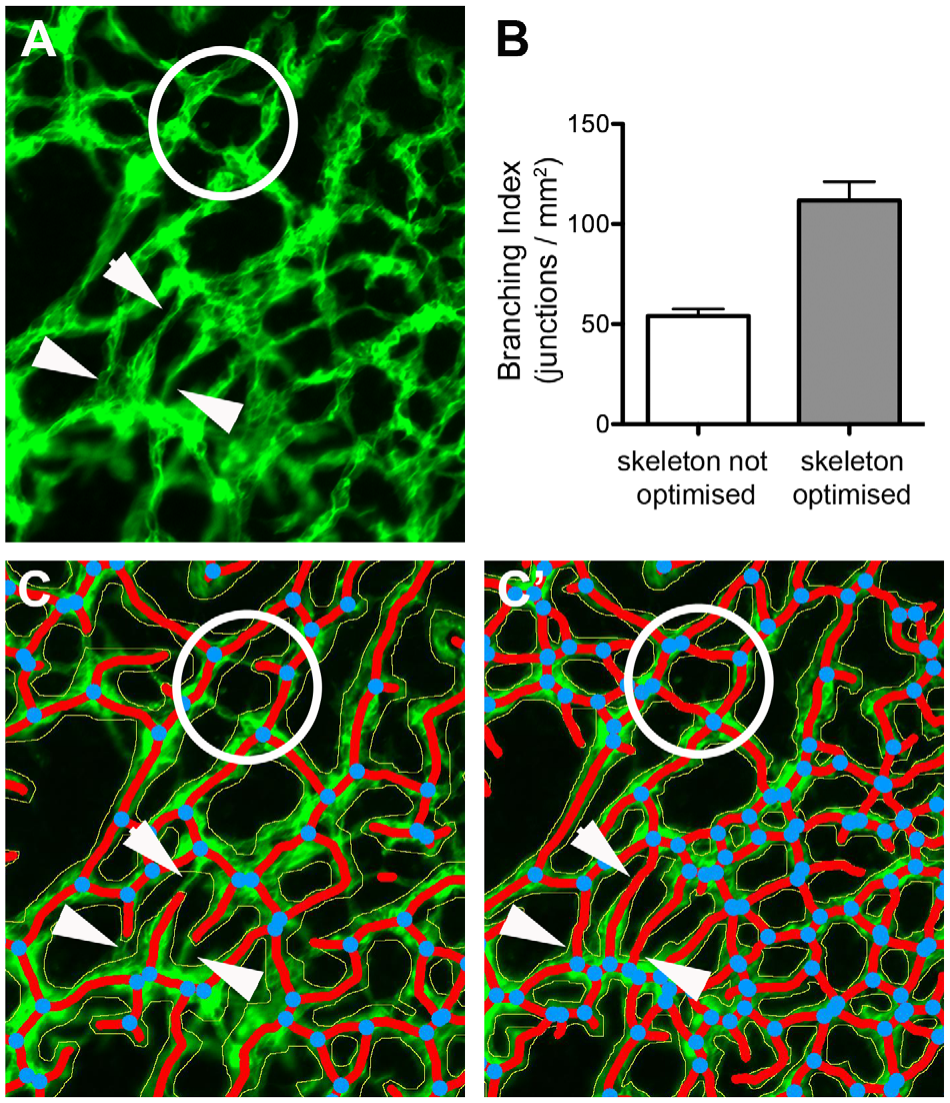
Optimising skeletons for a thorough analysis. The control allantois explants for the experiment shown in Fig. 5 were overlaid according to the pre-set parameters (skeletons not optimised) or after careful optimisation. This lead to significantly different branching indices (B; p<0.001, Mann Whitney T-test). (A) shows an enlarged area of a stained explant; the same area is shown after skeletonisation using the pre-set parameters (C) or after optimisation (C'. A few examples of missed vessels and junctions in the non-optimised skeleton are highlighted by ringed areas and arrowheads.

To ascertain the reproducibility of the results obtained, we routinely compared analyses performed by different users. Trends and ratios in the results obtained from user independent analysis were consistent throughout, demonstrating the robustness of the software. In Figure 6 we plotted the analysis images of the LY294002 and DMSO treated allantois explants carried out independently by two of the authors to illustrate this point. Whilst inhibitor treatment had a very significant effect on the parameters analysed, the investigator did not. It ought to be stressed, however, that the consistency of the values obtained in the analysis depends on the careful optimisation of the skeleton at the initial stage of the analysis. Figure 7 demonstrates this point, showing a stained fragment of a control allantois explant (A) together with skeletons generated with the non-optimised parameters (C) or after optimisation (C'). Careful optimisation of all four parameters allowed the detection of faint and thin vessels and junctions (C'), resulting in significantly different branching indices obtained (B). This underlines the importance of establishing clear morphological criteria prior to engaging in the automated assessment of vascular networks with AngioTool.

## Design and Implementation

AngioTool is written in Java and leverages the open source ImageJ (image processing and analysis; http://rsbweb.nih.gov/ij/) and several ImageJ plugins, the Mines Java Toolkit (http://inside.mines.edu/~dhale/jtk/index.html), Apache POI (Excel compatibility; http://poi.apache.org/), and JIDE Common Layer (Swing components; http://www.jidesoft.com/) libraries. AngioTool is open source and is distributed as a Windows executable.

8, or 16 bit greyscale and 24-bit colour images, 1.0 pixel aspect ratio, displaying labelled vasculature on a dark background are compatible with AngioTool. Segmentation of the vessel profiles is achieved using a fast multiscale Hessian-based enhancement filter [25], [26], [27]. Images are first convolved with fast recursive Gaussian kernel and tube-like structures and then computed based on a combination of the eigenvalues of the 2D Hessian matrix. The vesselness response is computed for a set of scales (sigmas, which denote the standard width of the Gaussian filter and is interpreted as vessel diameter in the GUI) chosen by the user. After segmentation, vessels are skeletonized [28] and analyzed (implementation based on [29]). Several morphometric parameters are computed including total and average vessel length, branching point density, and vascular density (Table 1). Some metrics are normalized to the area of the convex hull containing the region covered by the vessels allowing for comparison of differently sized vascular networks. Additionally, a fast box counting algorithm has been implemented for computation of lacunarity, an index for vascular structural nonuniformity [30], which is reported as the average lacunarity over all box sizes. AngioTool implements new concurrency features included in Java 7 to speed up computation-intensive tasks allowing for real-time analysis of vascular networks in multicore systems. Also, analysis time is significantly reduced as the number of central processing units in the system being used increases.

**Table 1:** Summary of angiogenesis related parameters computed by AngioTool.

Parameter	Description
Explant area	The area occupied by the convex hull containing the vessels in the image
Vessels area	The area of the segmented vessels
Vessel density	The percentage of area occupied by vessels inside the explant area
Total number of junctions	The total number of vessel junctions in the image
Branching index	The number of vessel junctions normalized per unit area
Total vessel* length	The sum of Euclidean distances between the pixels of all the vessels in the image
Average vessel length	Mean length of all the vessels in the image
Total number of endpoints	The number of open ended segments
Lacunarity	Mean lacunarity over all size boxes

*A vessel is defined after segmentation as a segment between two branching points or a branching point and an end point.

The images reported in this study are provided as test images together with a quick operation guide at AngioTool's website site.

## Availability and Future Directions

AngioTool is open source and can be downloaded at http://angiotool.nci.nih.gov. AngioTool's installation file is distributed as a windows executable. The installation process which is guided by a self-explanatory step-by-step wizard and instructions can be found at AngioTool's download website site. AngioTool has been tested in Windows 32-bit platforms, it is self-contained and only requires pre-installation of Java^TM^ 7.

Based on user feedback, future versions of AngioTool will include potential bug fixes and are expected to feature new metrics which may prove useful under certain experimental conditions, such as tube thickness and graphical and topographical analysis of vascular networks. New capabilities can also be added to the GUI to facilitate analysis such as zooming of the analysis window and reporting raw data on each vessel segment.

## Materials and Methods

### Ethics Statement

This work was conducted under the control of UK Home Office Certificate of Designation PCD80/4804 to the Babraham Research Campus and approved by the Babraham Institute institutional animal care committee (PPL 80/2335).

### Retinal angiogenesis

Postnatal P6 eyes were briefly fixed with 2% PFA in PBS at 4°C, then retinas were dissected and stored in methanol at -20°C. Immediately prior to staining retinas were re-fixed in 4% PFA for 20 minutes. PBS-washed retinas were stained with biotinylated isolectin B4 (Vectorlabs) followed by streptavidin-alexafluor 568 (Molecular Probes) and flat mounted for epifluorescence analysis on a Cell^R^ microscope (Olympus).

### Hindbrain angiogenesis

Hindbrains were dissected clean from surrounding tissue followed by fixation steps, dehydration and rehydration steps as described [3], prior to being whole-mount stained using a rat anti endomucin antibody (Santa Cruz Biotechnology). Samples were analysed from the ventricular side. Several overlapping photographs were taken of each hindbrain imaged using an epifluorescence microscope (Cell^R^, Olympus). Images were assembled using Photoshop software.

### Allantois explant

Allantoises were dissected from E8.5 embryos and cultured for 18 hours in 8-well µ-slides (Ibidi) coated with bovine fibronectin (Sigma) in DMEM (Gibco) supplemented with 10% fetal calf serum (Invitrogen) in a humidified tissue culture incubator at 37°C and 5% CO_2_ (Sanyo). Inhibitors were made up as per manufacturer's instructions and added into the growth medium when setting up the culture. Control explants were vehicle treated. Inhibitors were obtained from Calbiochem and used at the following concentrations: LY294002, 10 µM; Y-27632, 10 µM. Allantois explants were fixed with 4% paraformaldehyde, followed by wholemount staining with rat anti-VE-cadherin antibody (BD Biosciences) together with rat anti-endomucin. Overlapping pictures of the explants were taken with an inverted epifluorescence microscope (Olympus Cell^R^), making sure that no area was over-or underexposed. Photographs were assembled using the panorama function of Photoshop software.

## Supporting Information

Figure S1 **Microscopic hindbrain image used for analysis shown in** **Figure 2** **.**
(TIF)

Figure S2 **Microscopic hindbrain image used for analysis shown in** **Figure 2** **.**
(TIF)

Figure S3 **Microscopic hindbrain image used for analysis shown in** **Figure 2** **.**
(TIF)

Figure S4 **Microscopic retina image used for analyses shown in** **Figure 3** **.**
(TIF)

Figure S5 **Microscopic retina image used for analyses shown in** **Figure 3** **.**
(TIF)

Figure S6 **Microscopic retina image used for analyses shown in** **Figure 3** **.**
(TIF)

Figure S7 **Microscopic retina image used for analyses shown in** **Figure 3** **.**
(TIF)

Figure S8 **Microscopic image of a DMSO treated control allantois explant used for analysis shown in** **Figures 4** **and** **6** **.**
(TIF)

Figure S9 **Microscopic image of a DMSO treated control allantois explant used for analysis shown in** **Figures 4** **and** **6** **.**
(TIF)

Figure S10 **Microscopic image of a DMSO treated control allantois explant used for analysis shown in** **Figures 4** **and** **6** **.**
(TIF)

Figure S11 **Microscopic image of a DMSO treated control allantois explant used for analysis shown in** **Figures 4** **and** **6** **.**
(TIF)

Figure S12 **Microscopic image of a DMSO treated control allantois explant used for analysis shown in** **Figures 4** **and** **6** **.**
(TIF)

Figure S13 **Microscopic image of a DMSO treated control allantois explant used for analysis shown in** **Figures 4** **and** **6** **.**
(TIF)

Figure S14 **Microscopic image of a DMSO treated control allantois explant used for analysis shown in** **Figures 4** **and** **6** **.**
(TIF)

Figure S15 **Microscopic image of a DMSO treated control allantois explant used for analysis shown in** **Figures 4** **and** **6** **.**
(TIF)

Figure S16 **Microscopic image of a LY294002 treated allantois explant used for analysis shown in** **Figures 4** **and** **6** **.**
(TIF)

Figure S17 **Microscopic image of a LY294002 treated allantois explant used for analysis shown in** **Figures 4** **and** **6** **.**
(TIF)

Figure S18 **Microscopic image of a LY294002 treated allantois explant used for analysis shown in** **Figures 4** **and** **6** **.**
(TIF)

Figure S19 **Microscopic image of a LY294002 treated allantois explant used for analysis shown in** **Figures 4** **and** **6** **.**
(TIF)

Figure S20 **Microscopic image of a LY294002 treated allantois explant used for analysis shown in** **Figures 4** **and** **6** **.**
(TIF)

Figure S21 **Microscopic image of a LY294002 treated allantois explant used for analysis shown in** **Figures 4** **and** **6** **.**
(TIF)

Figure S22 **Microscopic image of a LY294002 treated allantois explant used for analysis shown in** **Figures 4** **and** **6** **.**
(TIF)

Figure S23 **Microscopic image of a LY294002 treated allantois explant used for analysis shown in** **Figures 4** **and** **6** **.**
(TIF)

Figure S24 **Microscopic image of a water treated control allantois explant used for analysis shown in** **Figure 5** **.**
(TIF)

Figure S25 **Microscopic image of a water treated control allantois explant used for analysis shown in** **Figure 5** **.**
(TIF)

Figure S26 **Microscopic image of a water treated control allantois explant used for analysis shown in** **Figure 5** **.**
(TIF)

Figure S27 **Microscopic image of a water treated control allantois explant used for analysis shown in** **Figure 5** **.**
(TIF)

Figure S28 **Microscopic image of a water treated control allantois explant used for analysis shown in** **Figure 5** **.**
(TIF)

Figure S29 **Microscopic image of a water treated control allantois explant used for analysis shown in** **Figure 5** **.**
(TIF)

Figure S30 **Microscopic image of a water treated control allantois explant used for analysis shown in** **Figure 5** **.**
(TIF)

Figure S31 **Microscopic image of a Y27632 treated allantois explant used for analysis shown in** **Figure 5** **.**
(TIF)

Figure S32 **Microscopic image of a Y27632 treated allantois explant used for analysis shown in** **Figure 5** **.**
(TIF)

Figure S33 **Microscopic image of a Y27632 treated allantois explant used for analysis shown in** **Figure 5** **.**
(TIF)

Figure S34 **Microscopic image of a Y27632 treated allantois explant used for analysis shown in** **Figure 5** **.**
(TIF)

Figure S35 **Microscopic image of a Y27632 treated allantois explant used for analysis shown in** **Figure 5** **.**
(TIF)

Figure S36 **Microscopic image of a Y27632 treated allantois explant used for analysis shown in** **Figure 5** **.**
(TIF)

Figure S37 **Microscopic image of a Y27632 treated allantois explant used for analysis shown in** **Figure 5** **.**
(TIF)

Figure S38 **Microscopic image of a Y27632 treated allantois explant used for analysis shown in** **Figure 5** **.**
(TIF)

Figure S39 **Microscopic image of a Y27632 treated allantois explant used for analysis shown in** **Figure 5** **.**
(TIF)

Figure S40 **Microscopic image of a Y27632 treated allantois explant used for analysis shown in** **Figure 5** **.**
(TIF)
