# Supplementary figures and images for: A Computational Tool for Quantitative Analysis of Vascular Networks
Source: PLoS One. 2011 Nov 16;6(11):e27385. doi: 10.1371/journal.pone.0027385 (PMC3217985; doi:10.1371/journal.pone.0027385)

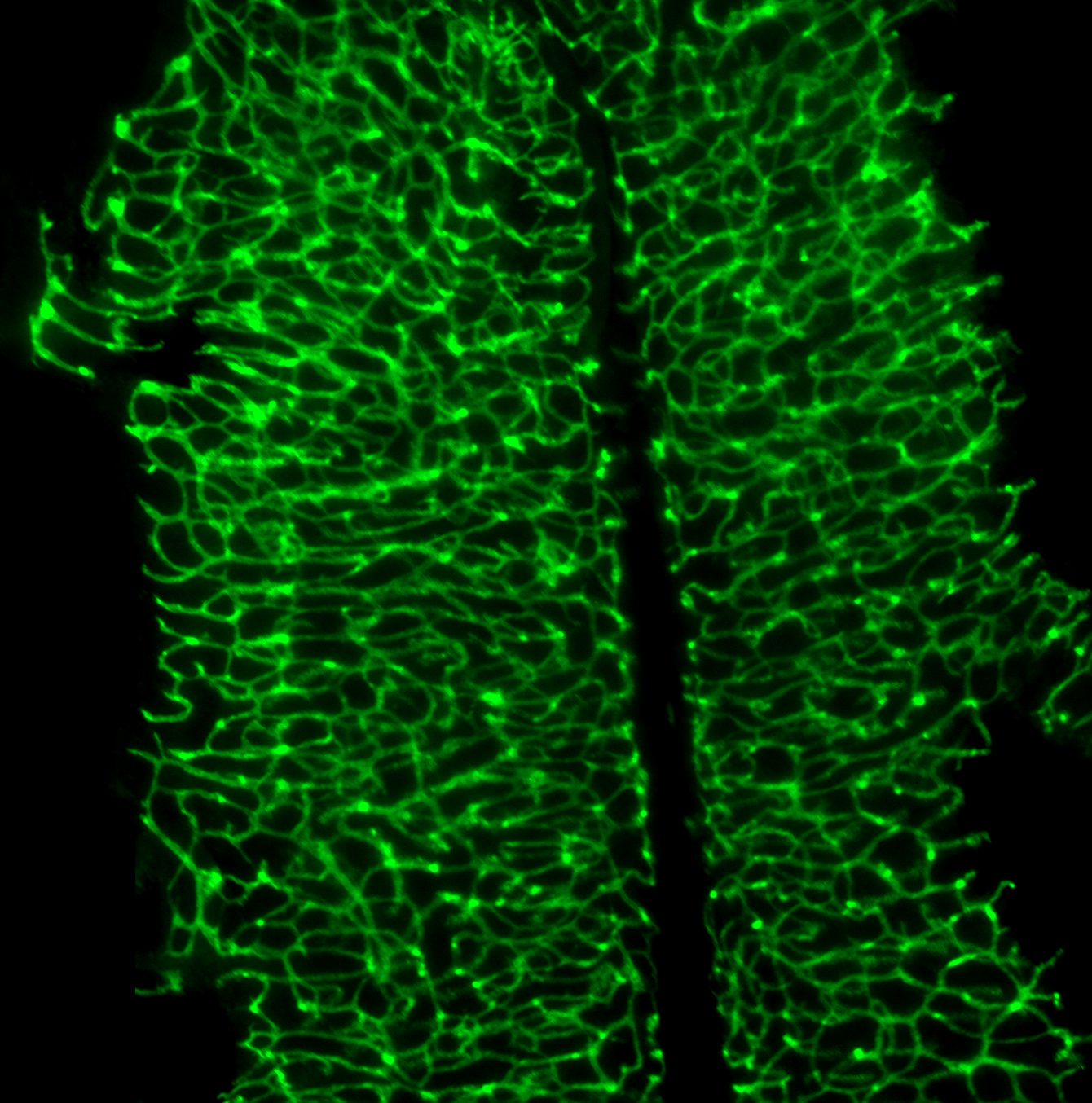

Supplement: Figure S1 — Microscopic hindbrain image used for analysis shown in Figure 2 . (TIF) [file pone.0027385.s001.tif]

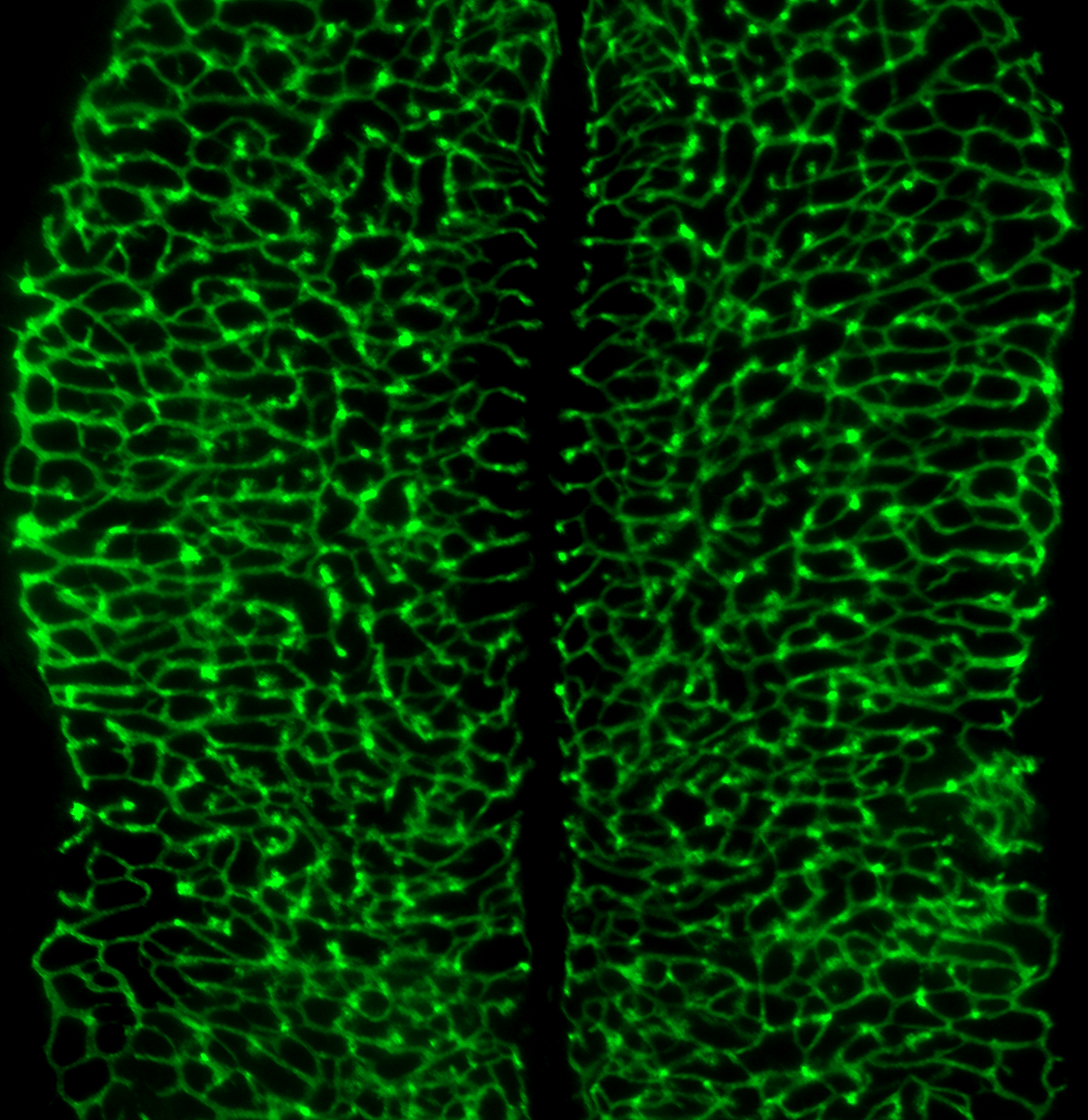

Supplement: Figure S2 — Microscopic hindbrain image used for analysis shown in Figure 2 . (TIF) [file pone.0027385.s002.tif]

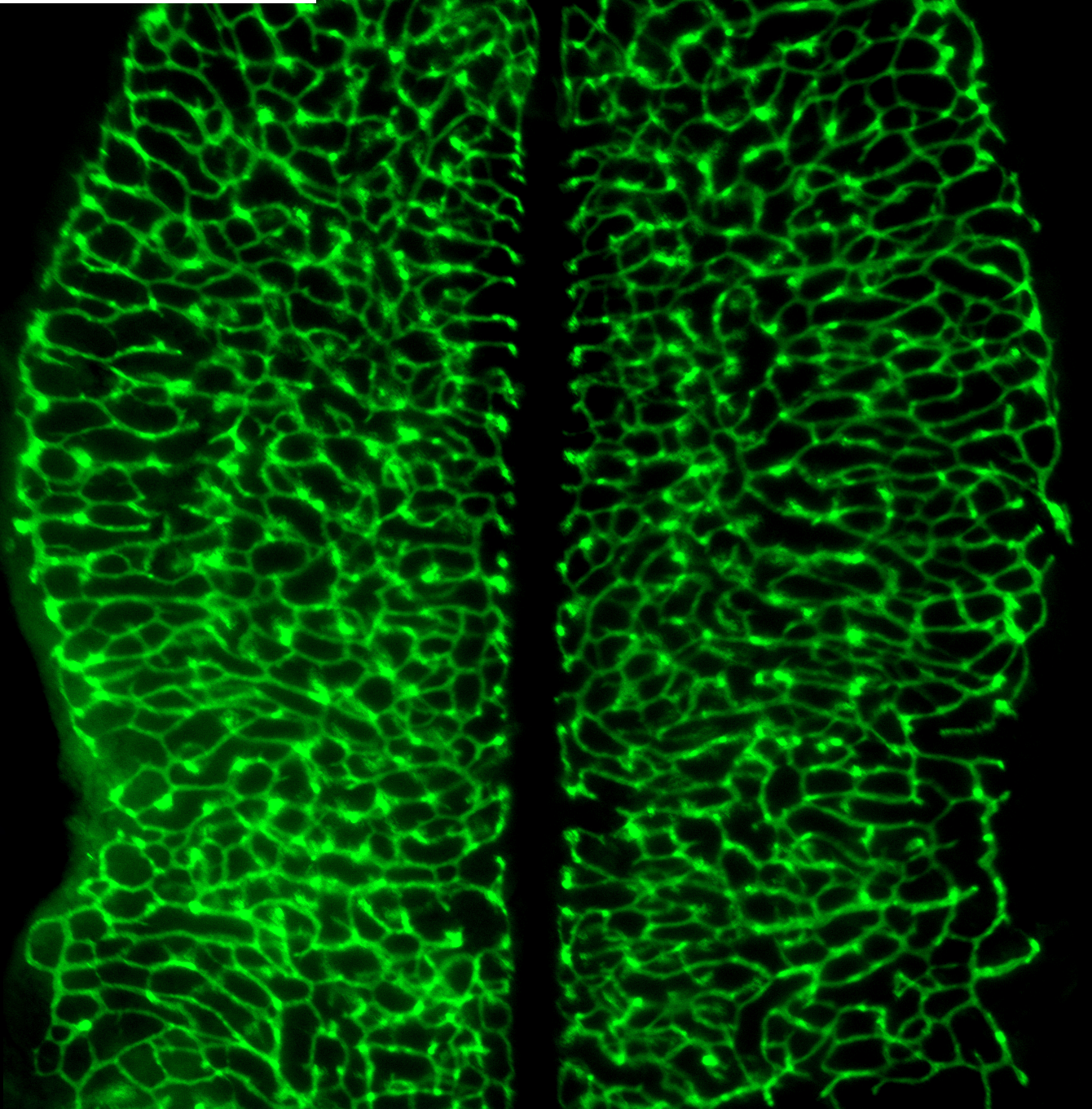

Supplement: Figure S3 — Microscopic hindbrain image used for analysis shown in Figure 2 . (TIF) [file pone.0027385.s003.tif]

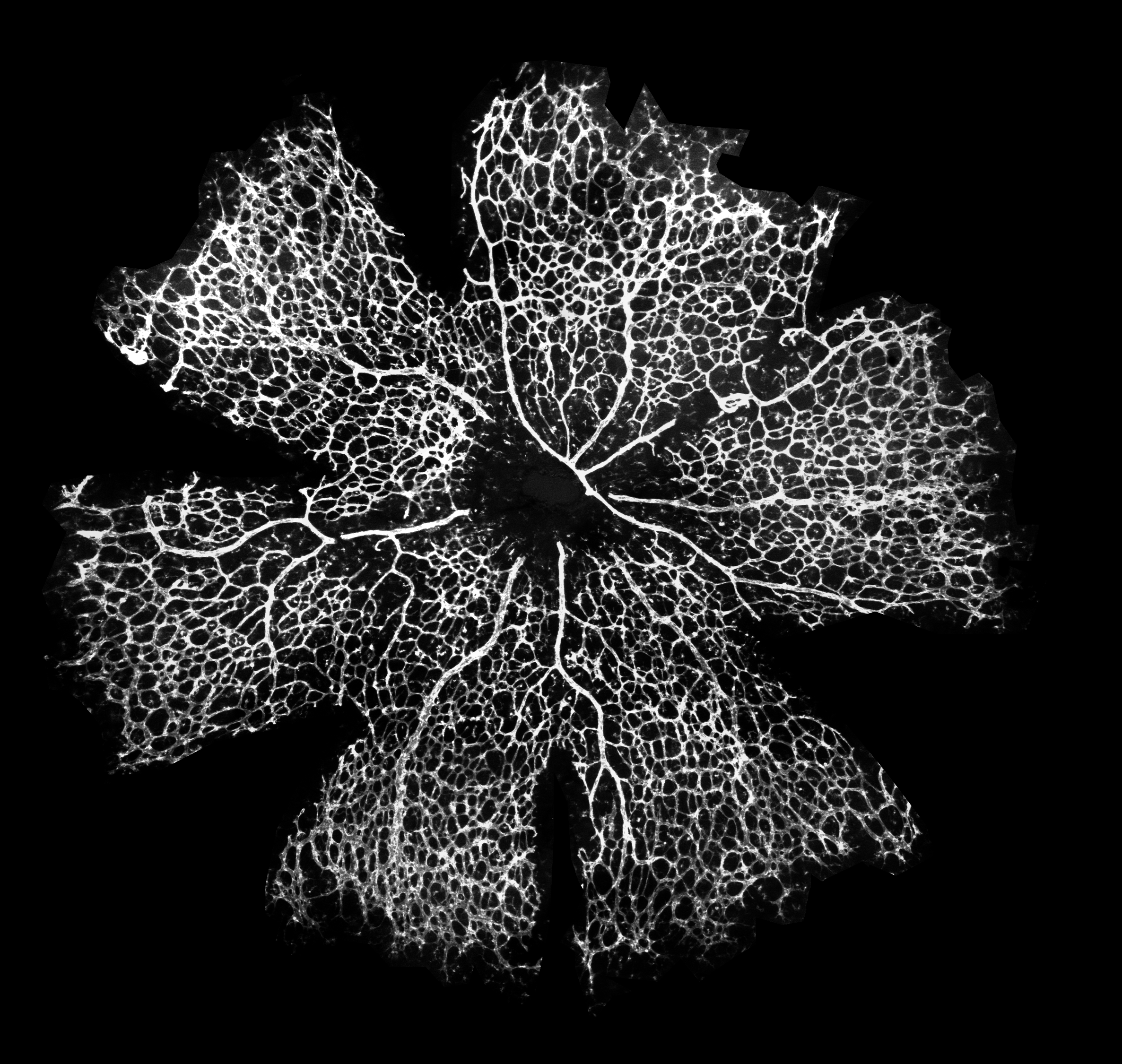

Supplement: Figure S4 — Microscopic retina image used for analyses shown in Figure 3 . (TIF) [file pone.0027385.s004.tif]

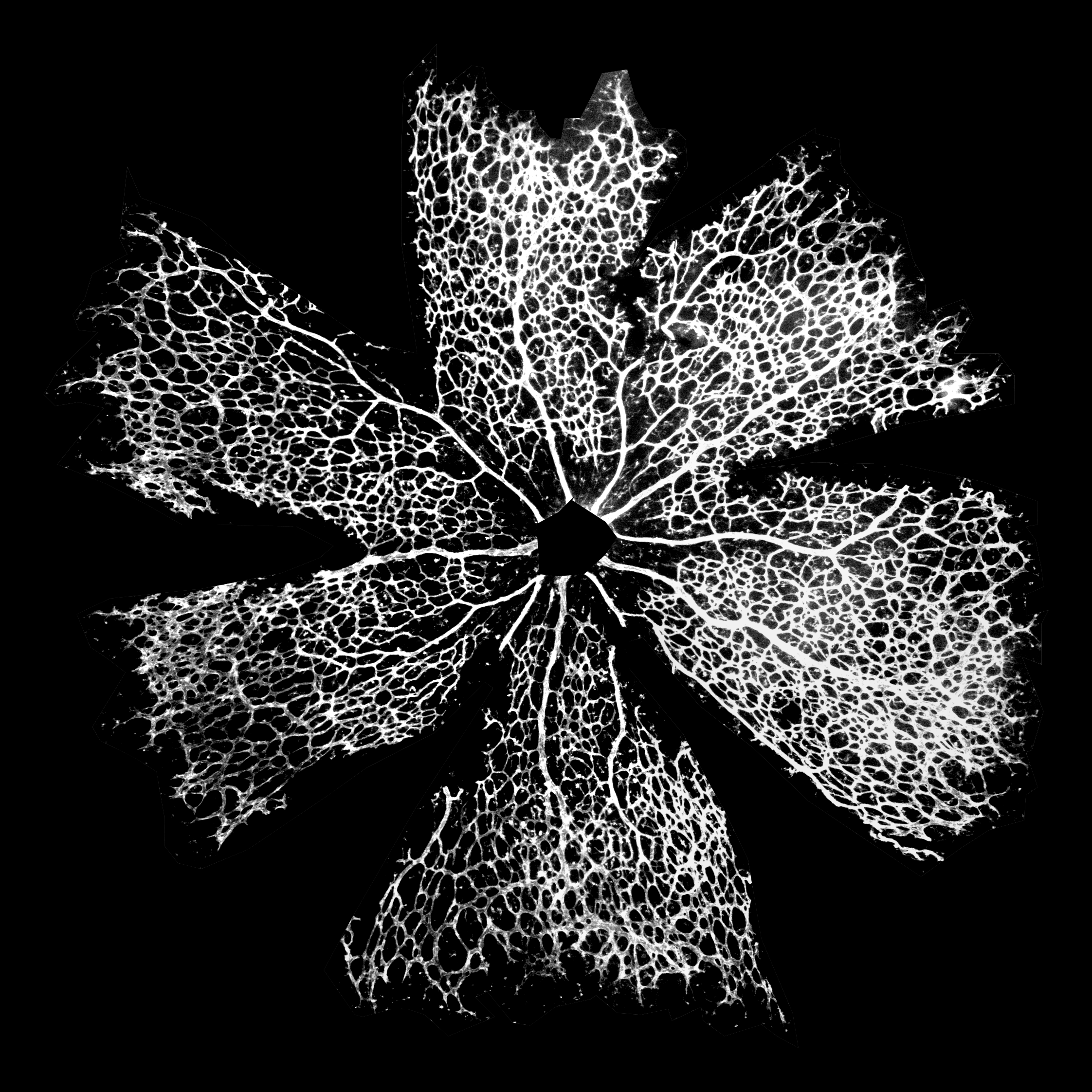

Supplement: Figure S5 — Microscopic retina image used for analyses shown in Figure 3 . (TIF) [file pone.0027385.s005.tif]

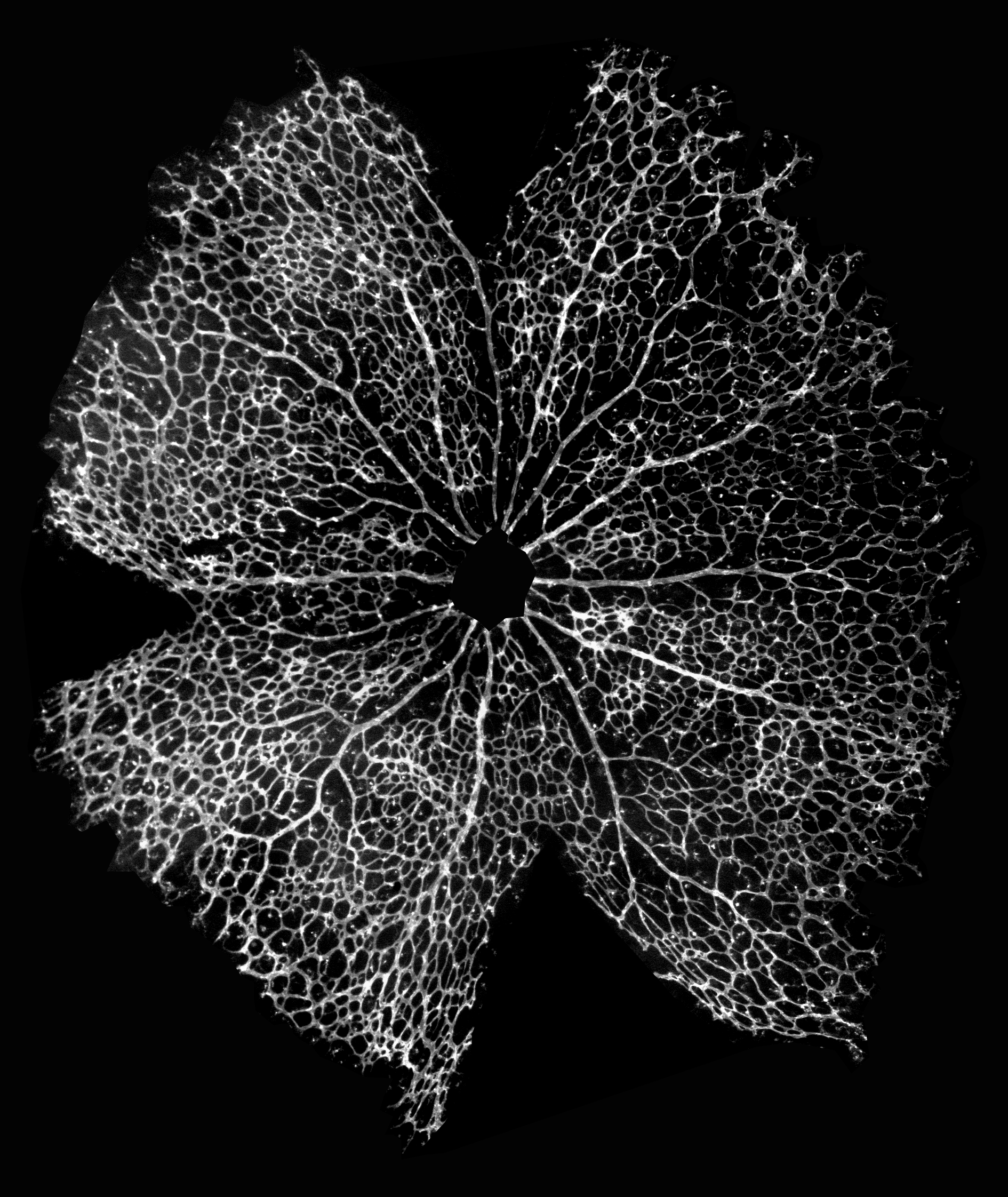

Supplement: Figure S6 — Microscopic retina image used for analyses shown in Figure 3 . (TIF) [file pone.0027385.s006.tif]

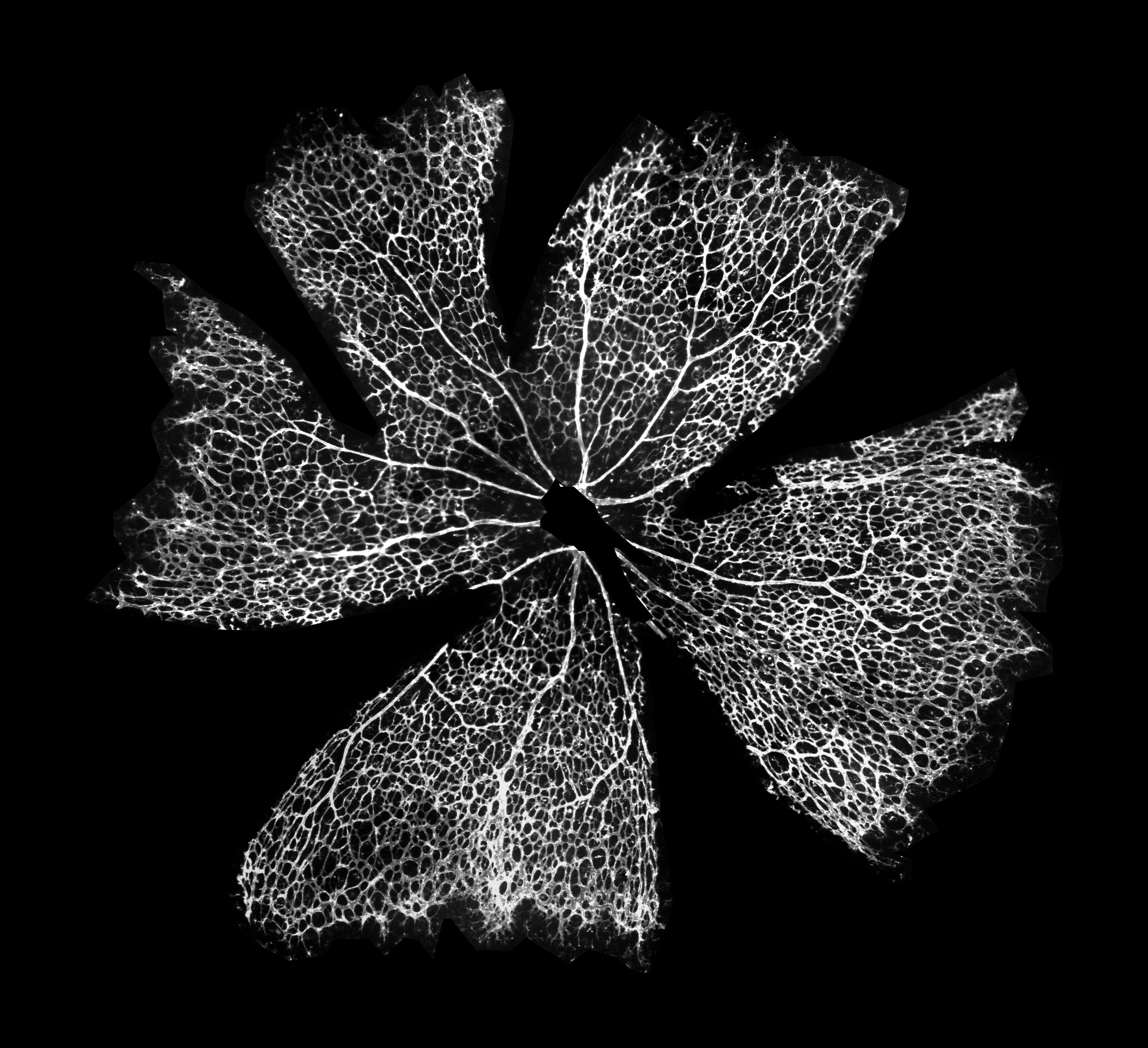

Supplement: Figure S7 — Microscopic retina image used for analyses shown in Figure 3 . (TIF) [file pone.0027385.s007.tif]

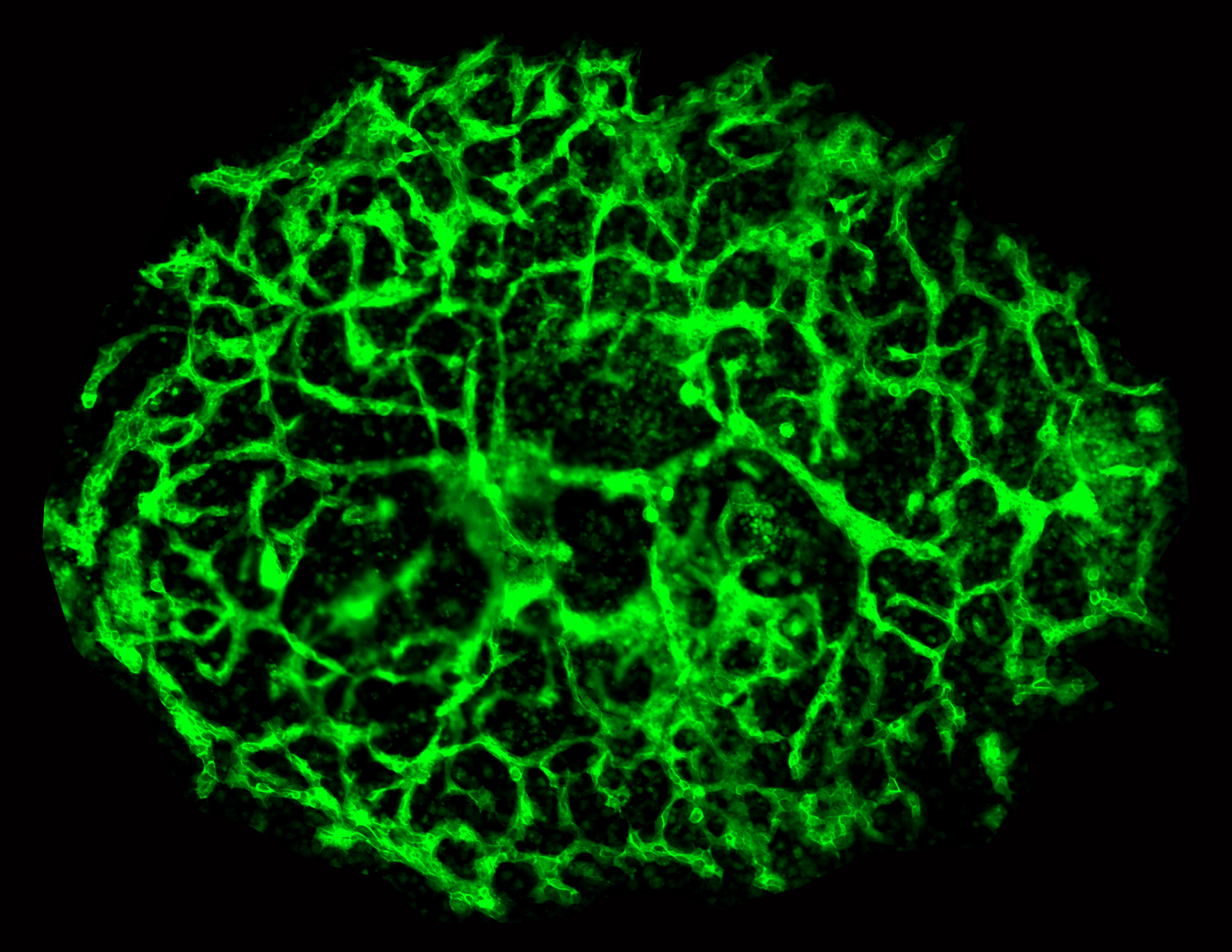

Supplement: Figure S8 — Microscopic image of a DMSO treated control allantois explant used for analysis shown in Figures 4 and 6 . (TIF) [file pone.0027385.s008.tif]

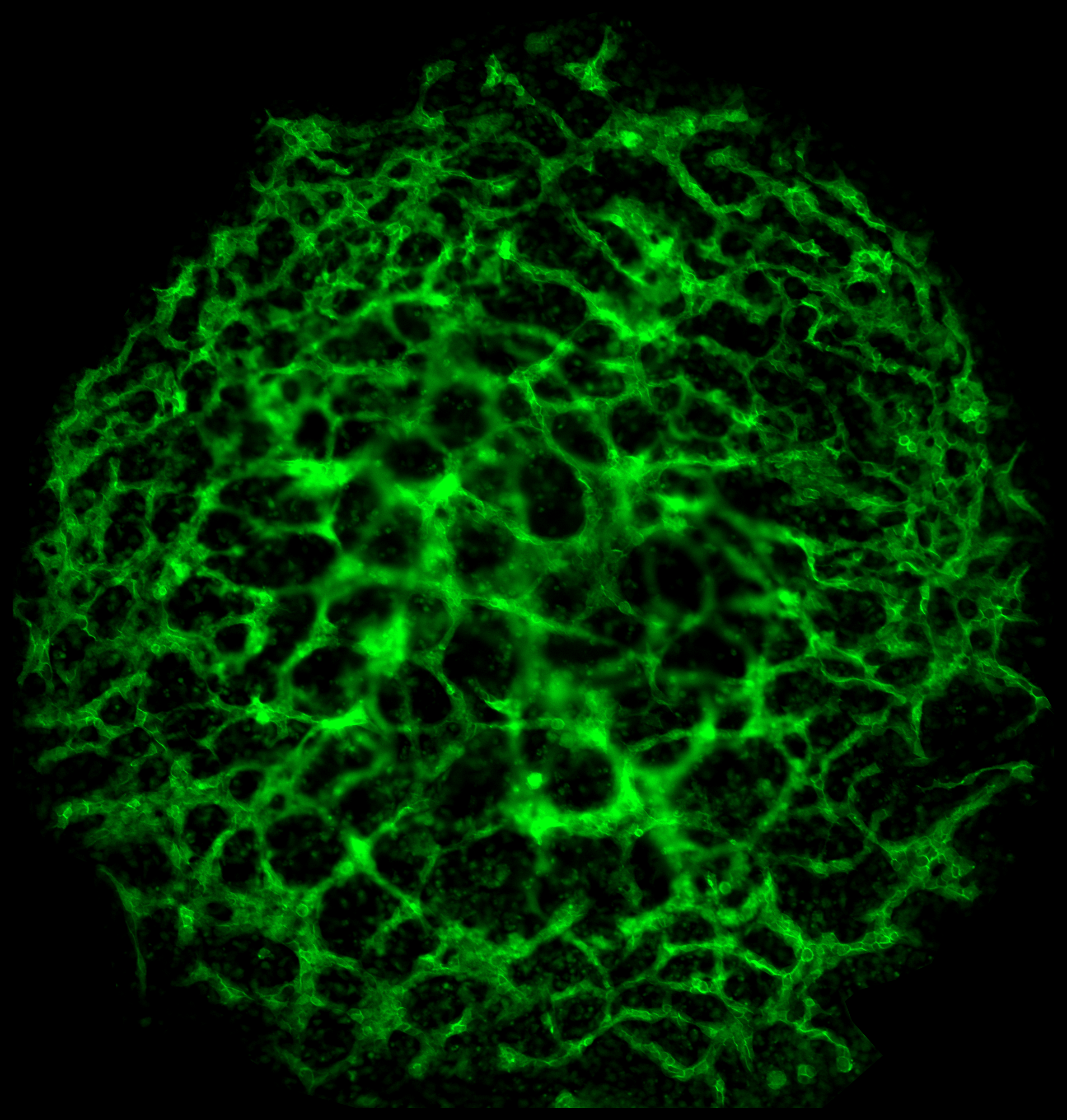

Supplement: Figure S9 — Microscopic image of a DMSO treated control allantois explant used for analysis shown in Figures 4 and 6 . (TIF) [file pone.0027385.s009.tif]

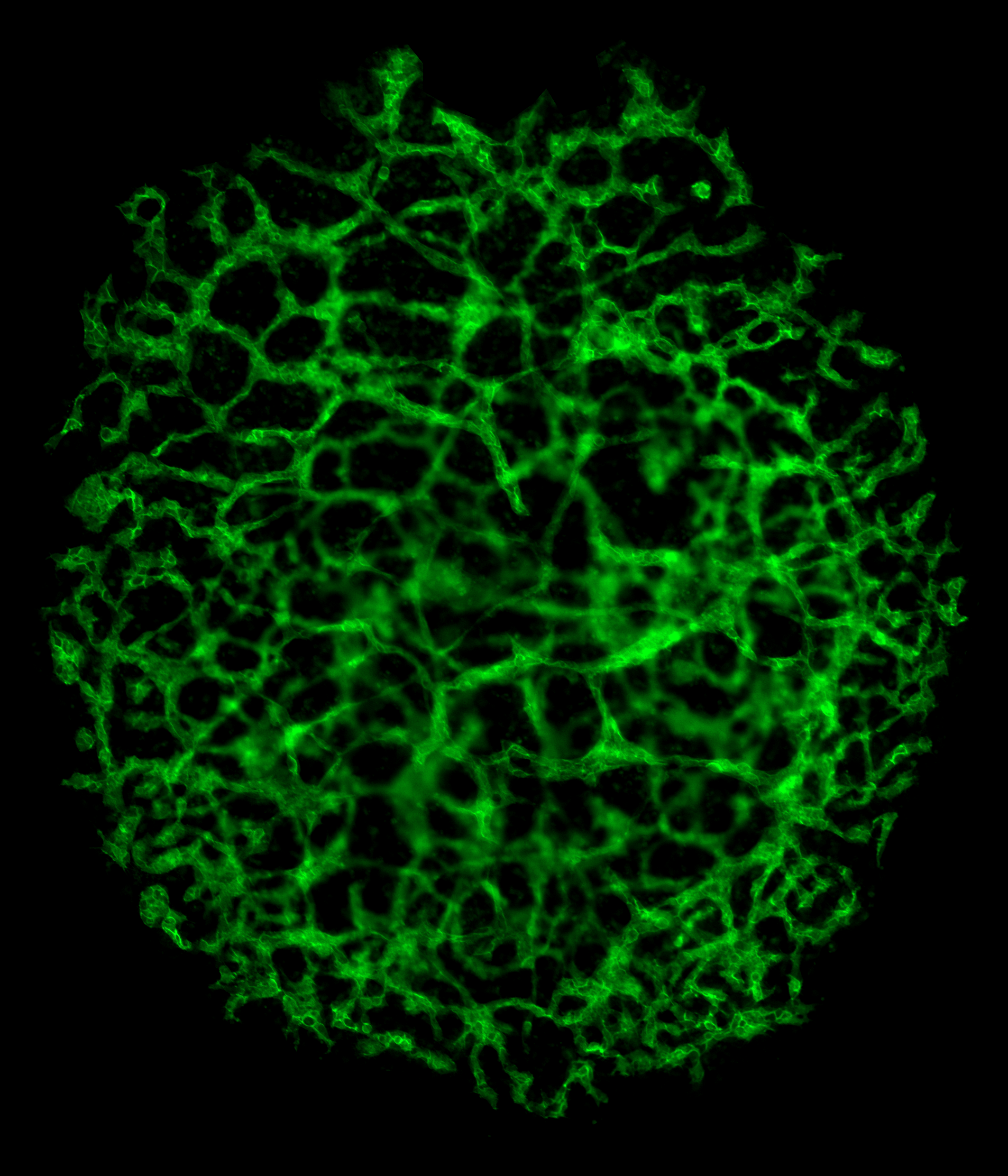

Supplement: Figure S10 — Microscopic image of a DMSO treated control allantois explant used for analysis shown in Figures 4 and 6 . (TIF) [file pone.0027385.s010.tif]

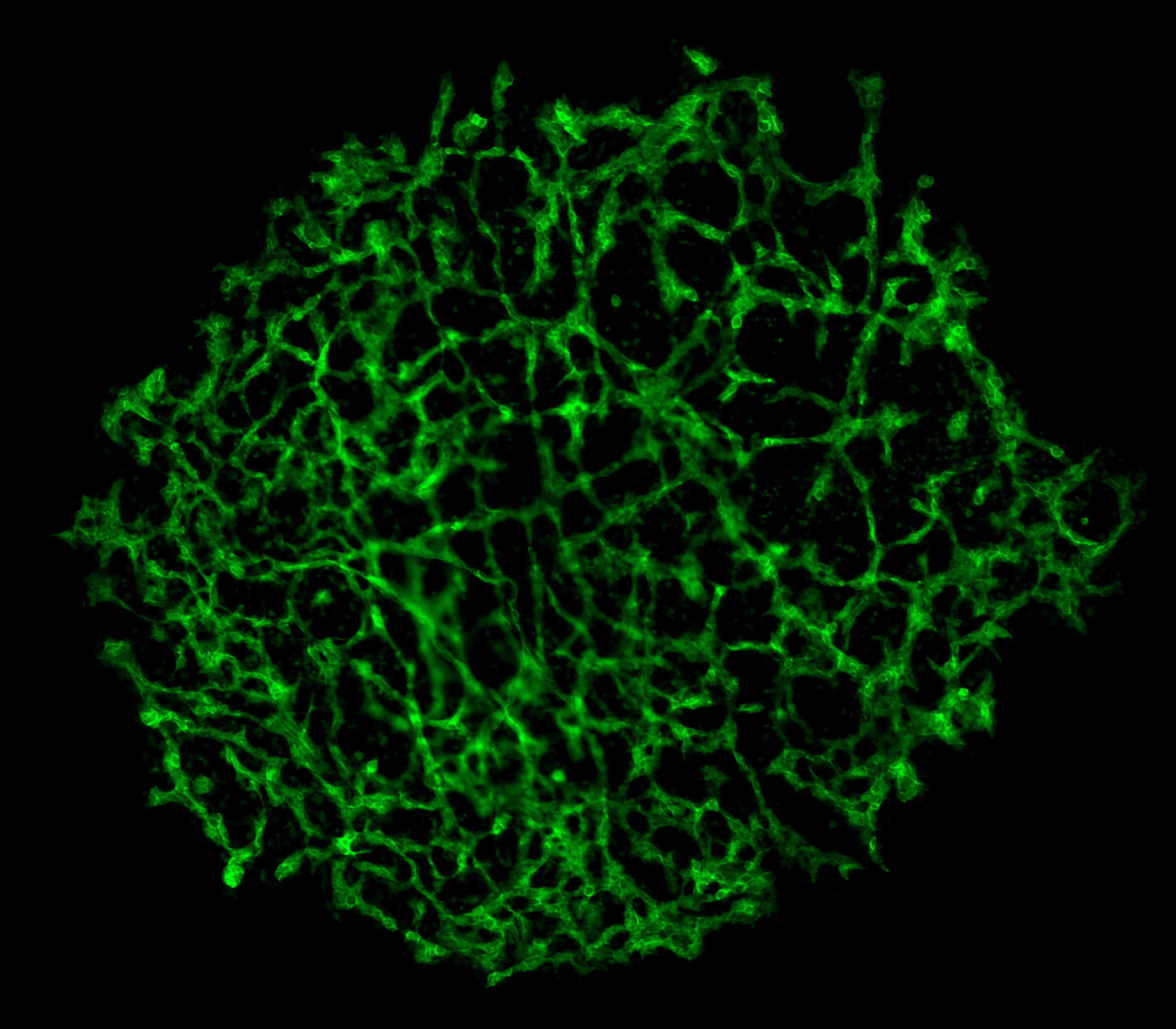

Supplement: Figure S11 — Microscopic image of a DMSO treated control allantois explant used for analysis shown in Figures 4 and 6 . (TIF) [file pone.0027385.s011.tif]

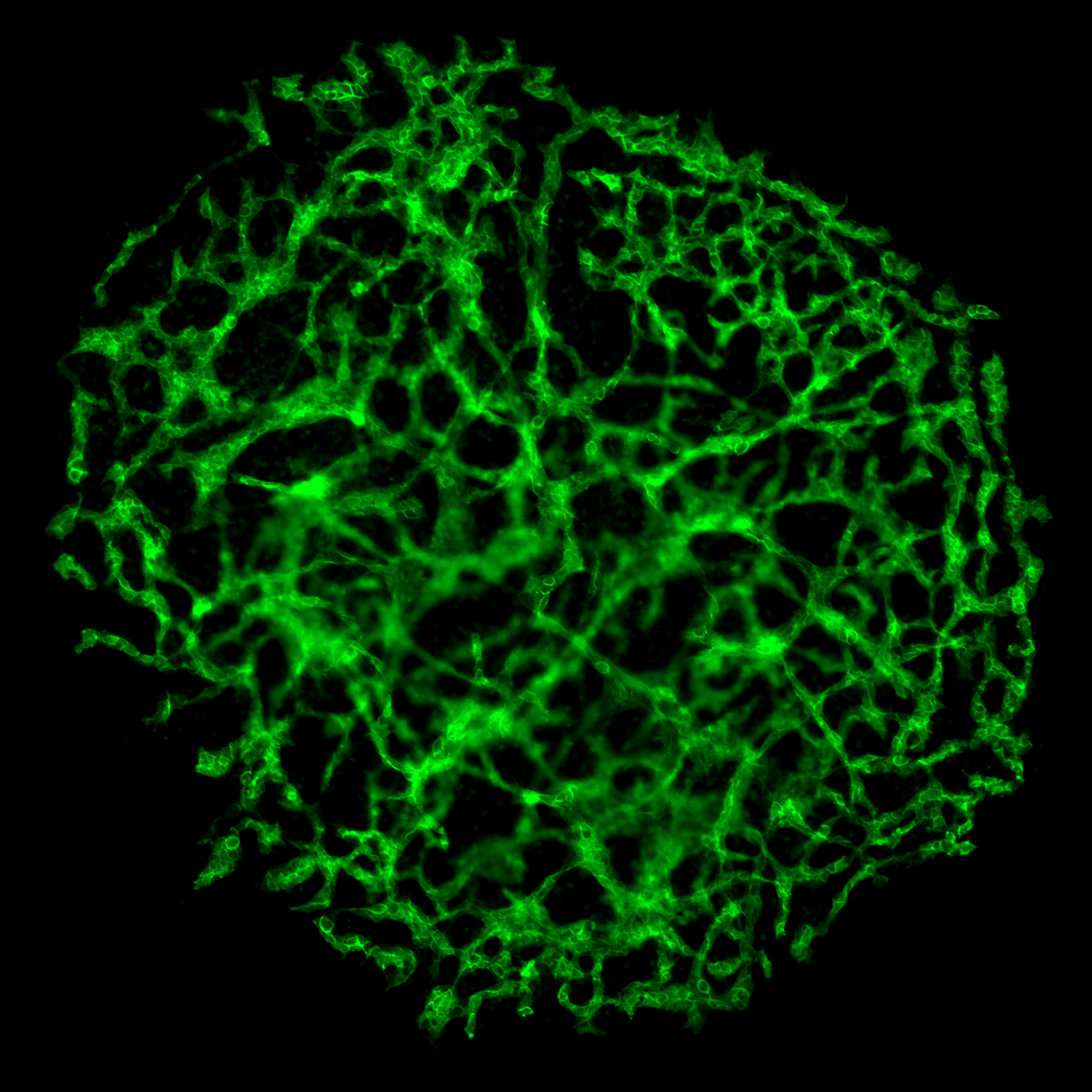

Supplement: Figure S12 — Microscopic image of a DMSO treated control allantois explant used for analysis shown in Figures 4 and 6 . (TIF) [file pone.0027385.s012.tif]

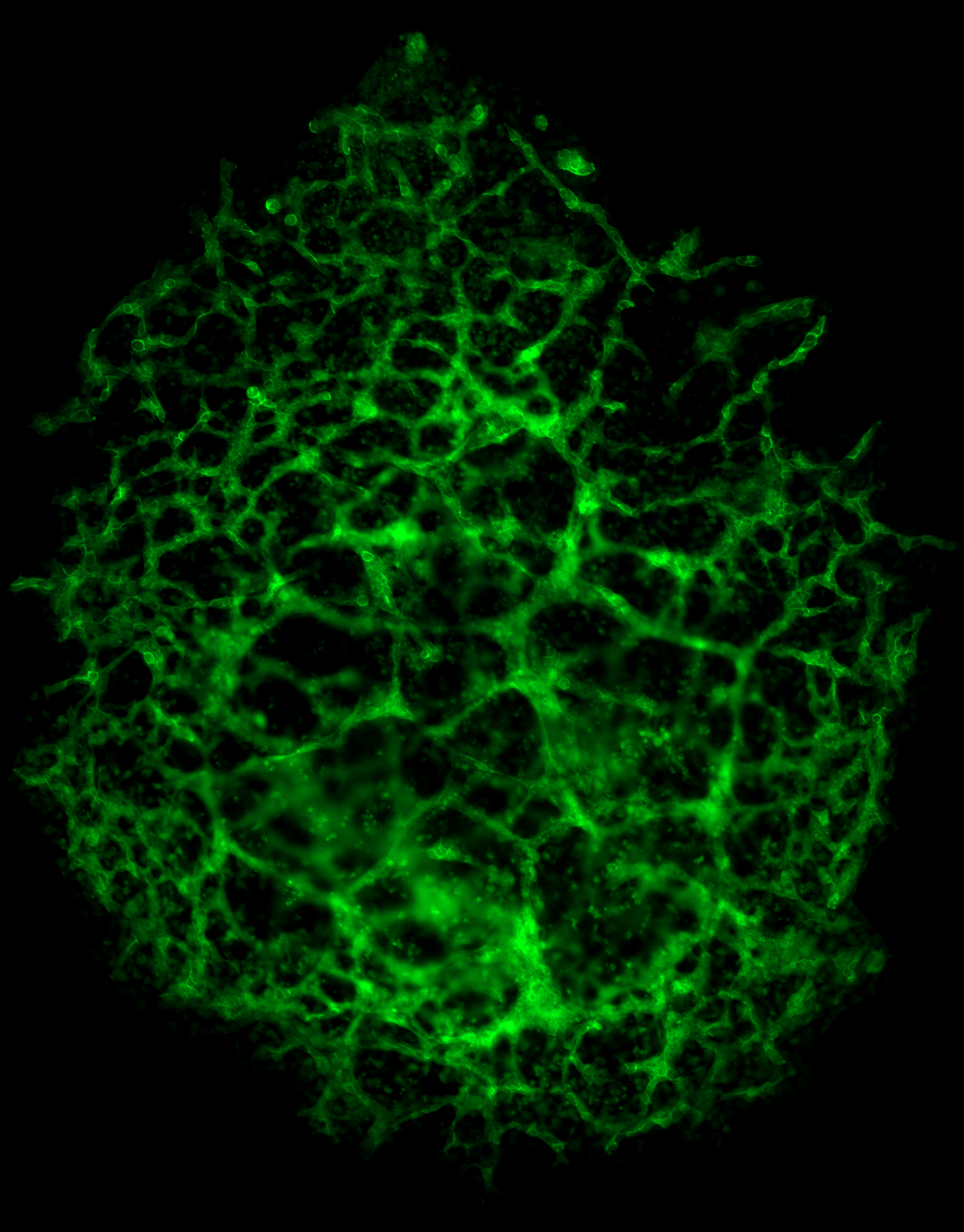

Supplement: Figure S13 — Microscopic image of a DMSO treated control allantois explant used for analysis shown in Figures 4 and 6 . (TIF) [file pone.0027385.s013.tif]

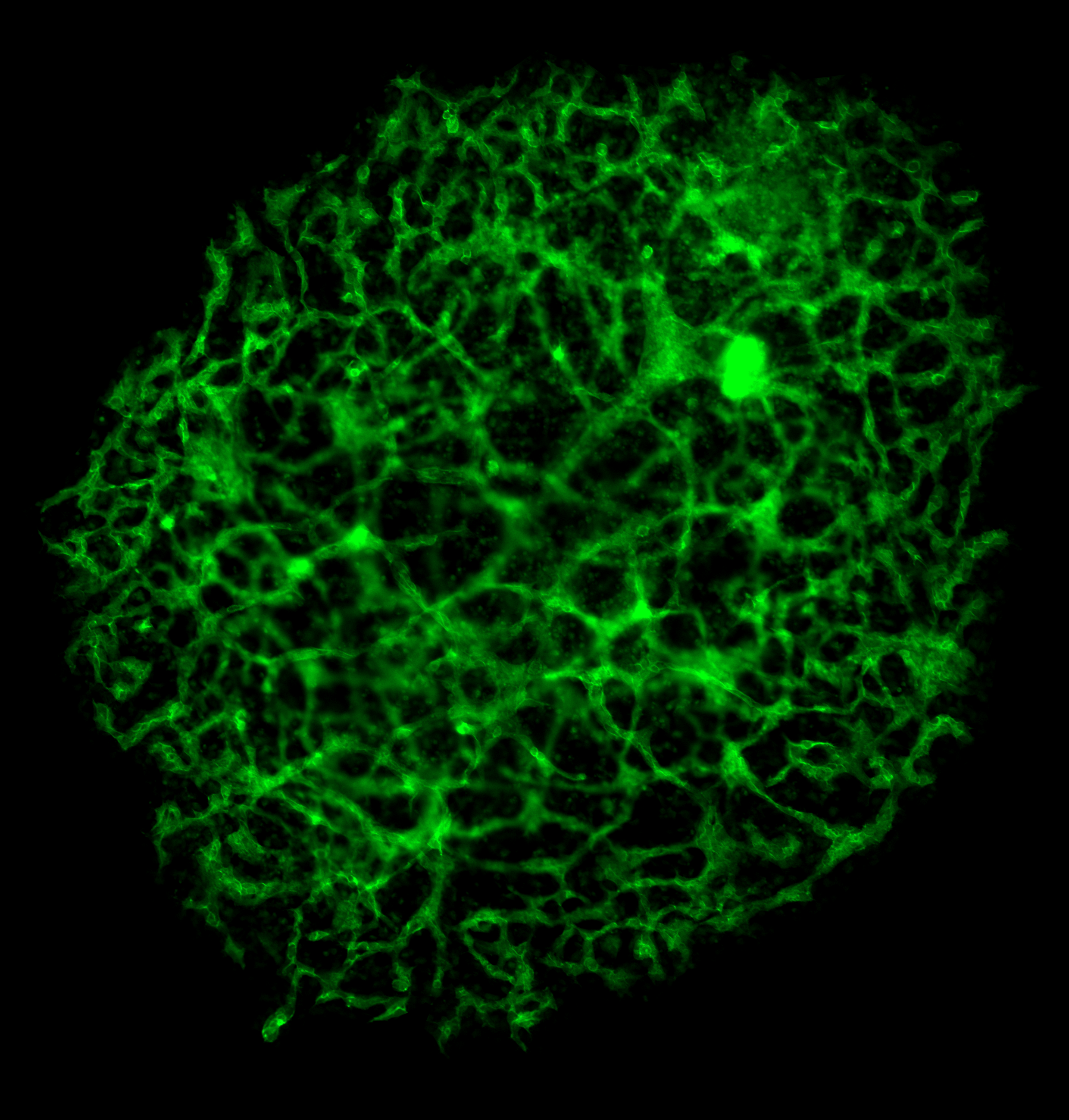

Supplement: Figure S14 — Microscopic image of a DMSO treated control allantois explant used for analysis shown in Figures 4 and 6 . (TIF) [file pone.0027385.s014.tif]

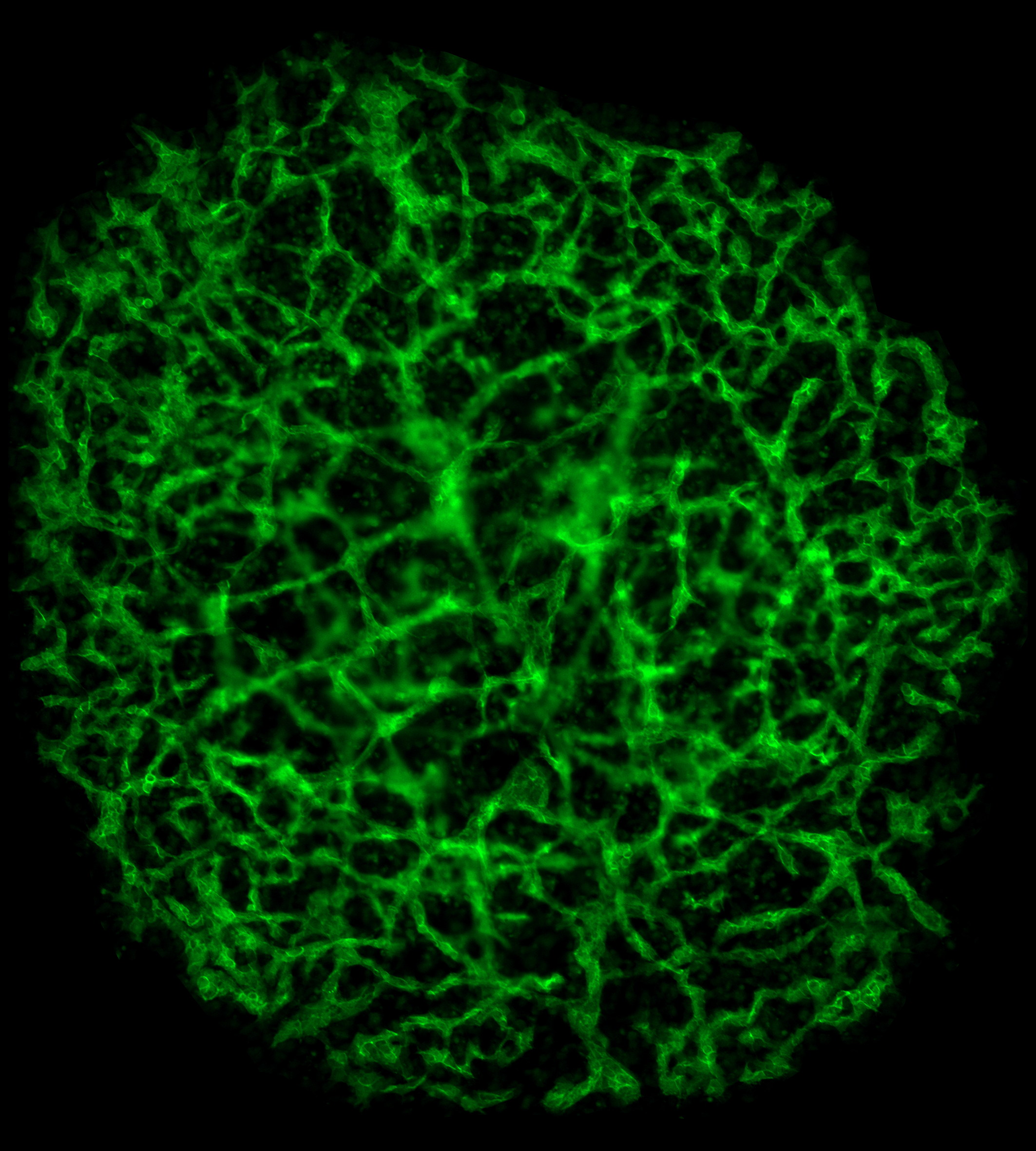

Supplement: Figure S15 — Microscopic image of a DMSO treated control allantois explant used for analysis shown in Figures 4 and 6 . (TIF) [file pone.0027385.s015.tif]

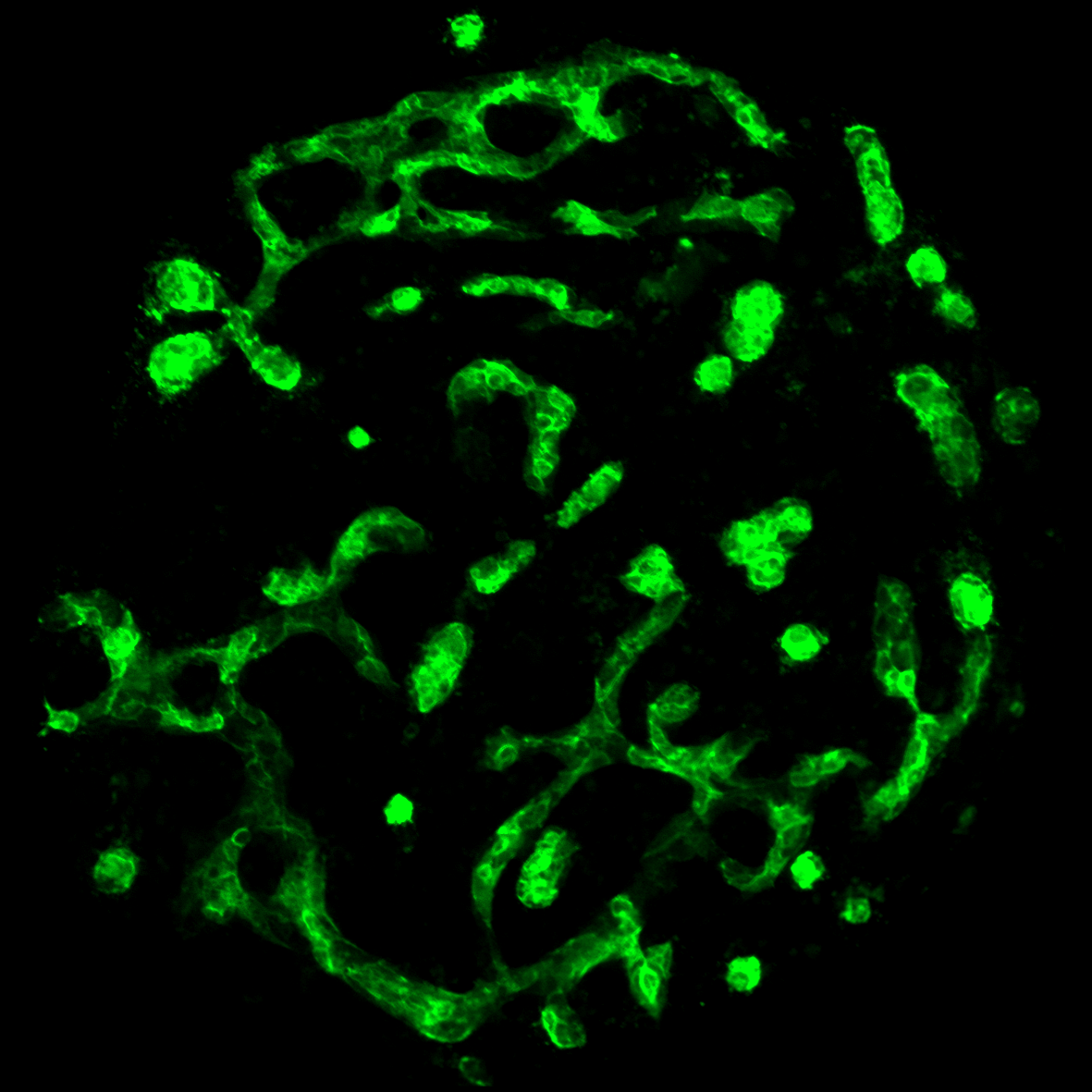

Supplement: Figure S16 — Microscopic image of a LY294002 treated allantois explant used for analysis shown in Figures 4 and 6 . (TIF) [file pone.0027385.s016.tif]

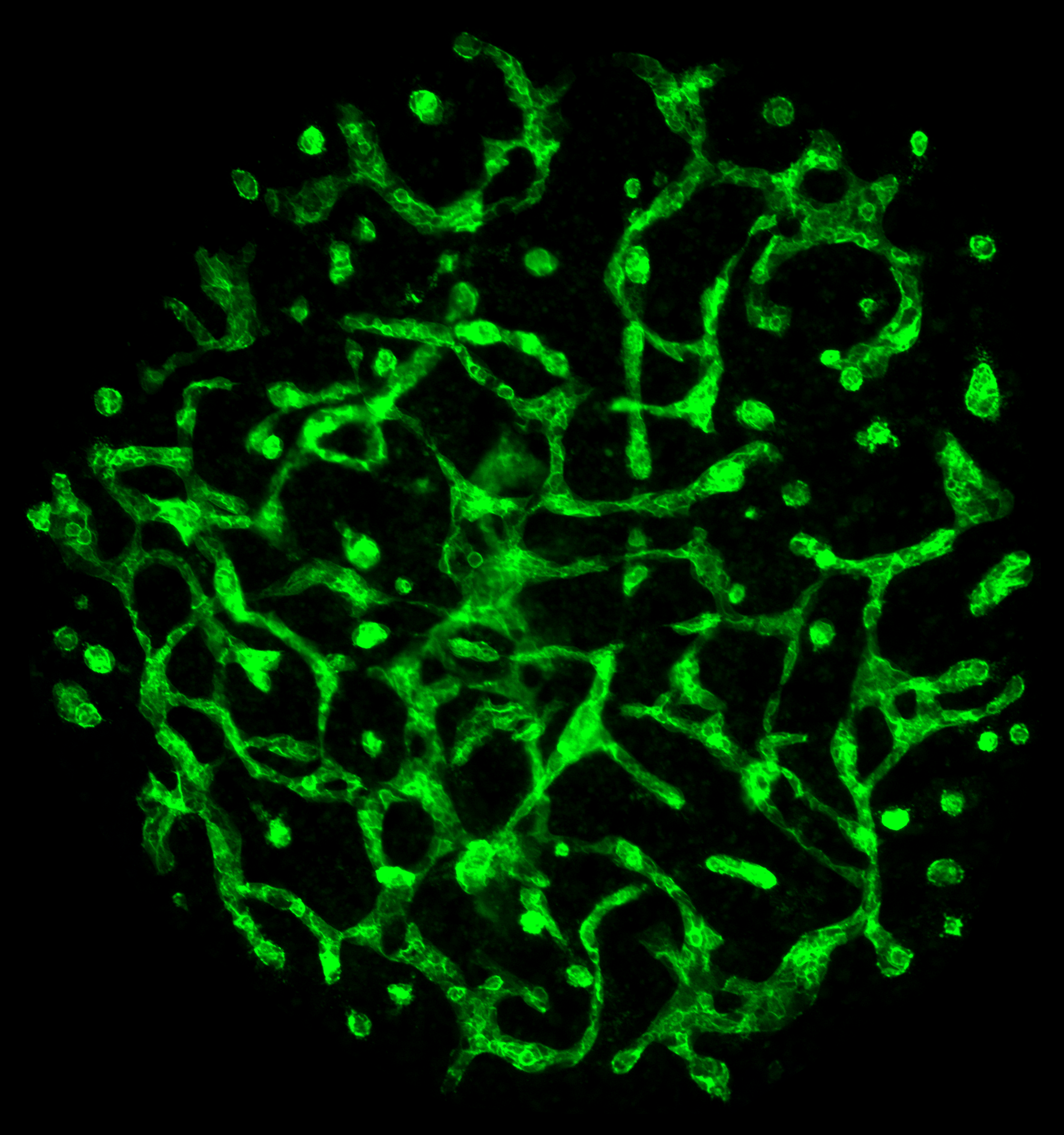

Supplement: Figure S17 — Microscopic image of a LY294002 treated allantois explant used for analysis shown in Figures 4 and 6 . (TIF) [file pone.0027385.s017.tif]

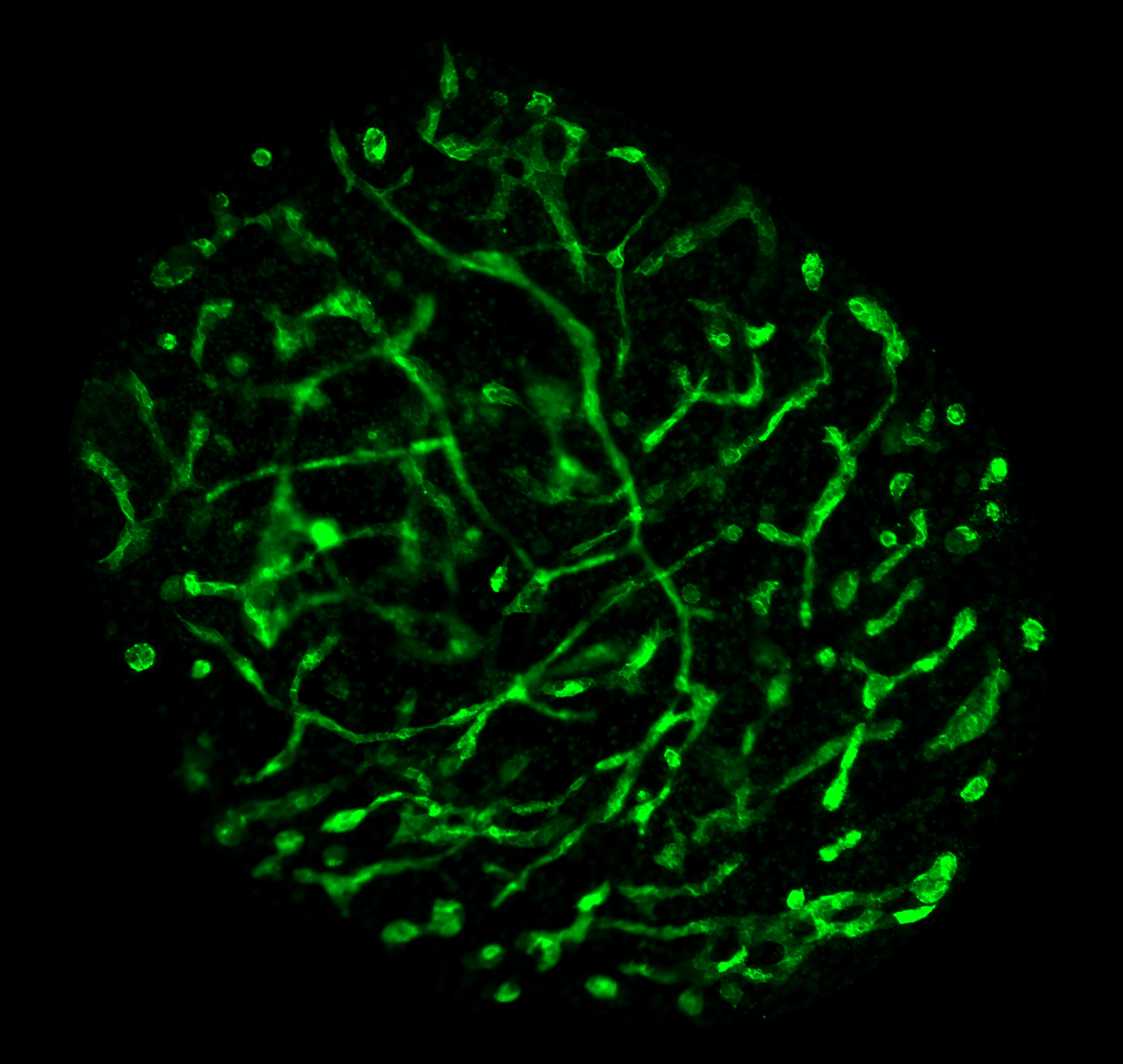

Supplement: Figure S18 — Microscopic image of a LY294002 treated allantois explant used for analysis shown in Figures 4 and 6 . (TIF) [file pone.0027385.s018.tif]

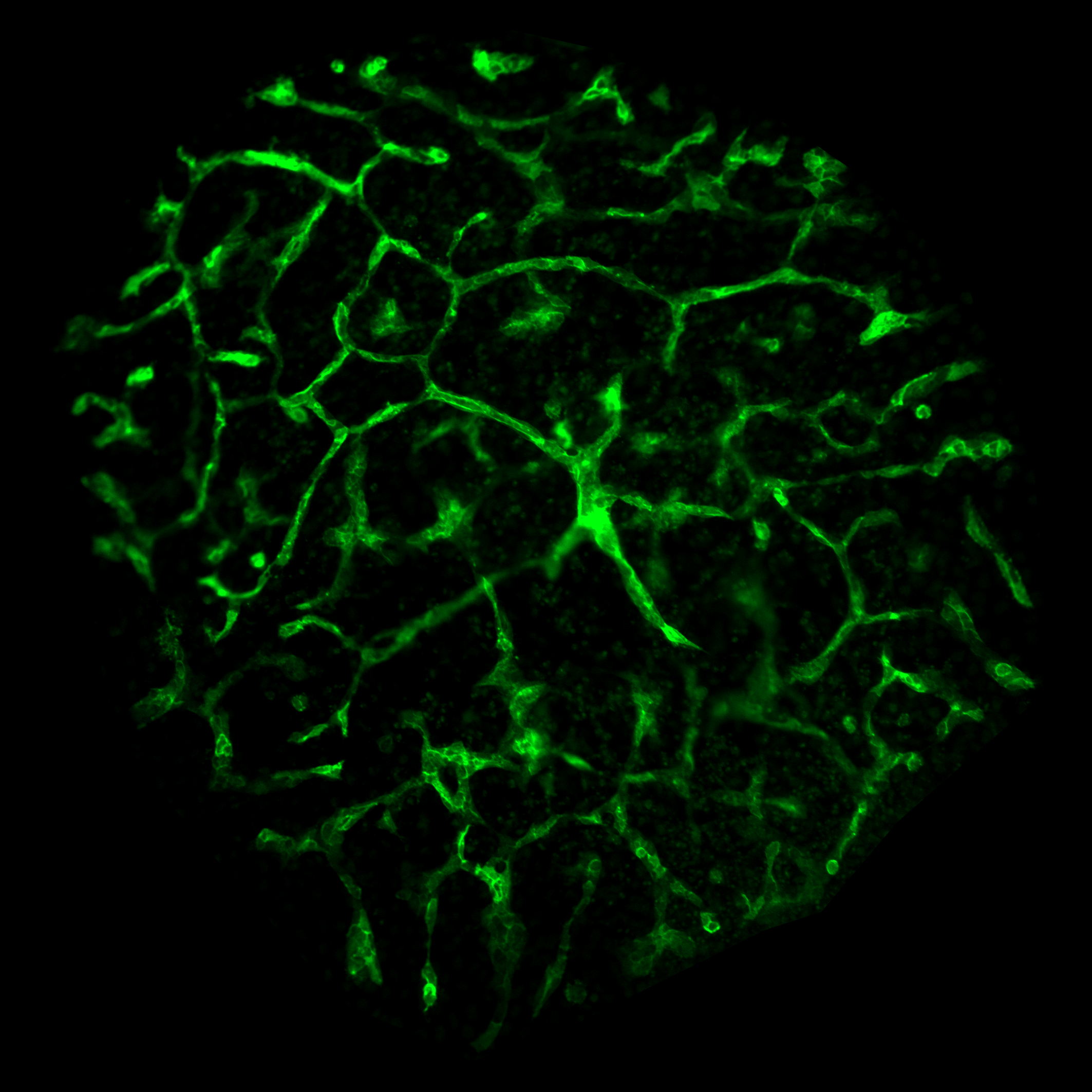

Supplement: Figure S19 — Microscopic image of a LY294002 treated allantois explant used for analysis shown in Figures 4 and 6 . (TIF) [file pone.0027385.s019.tif]

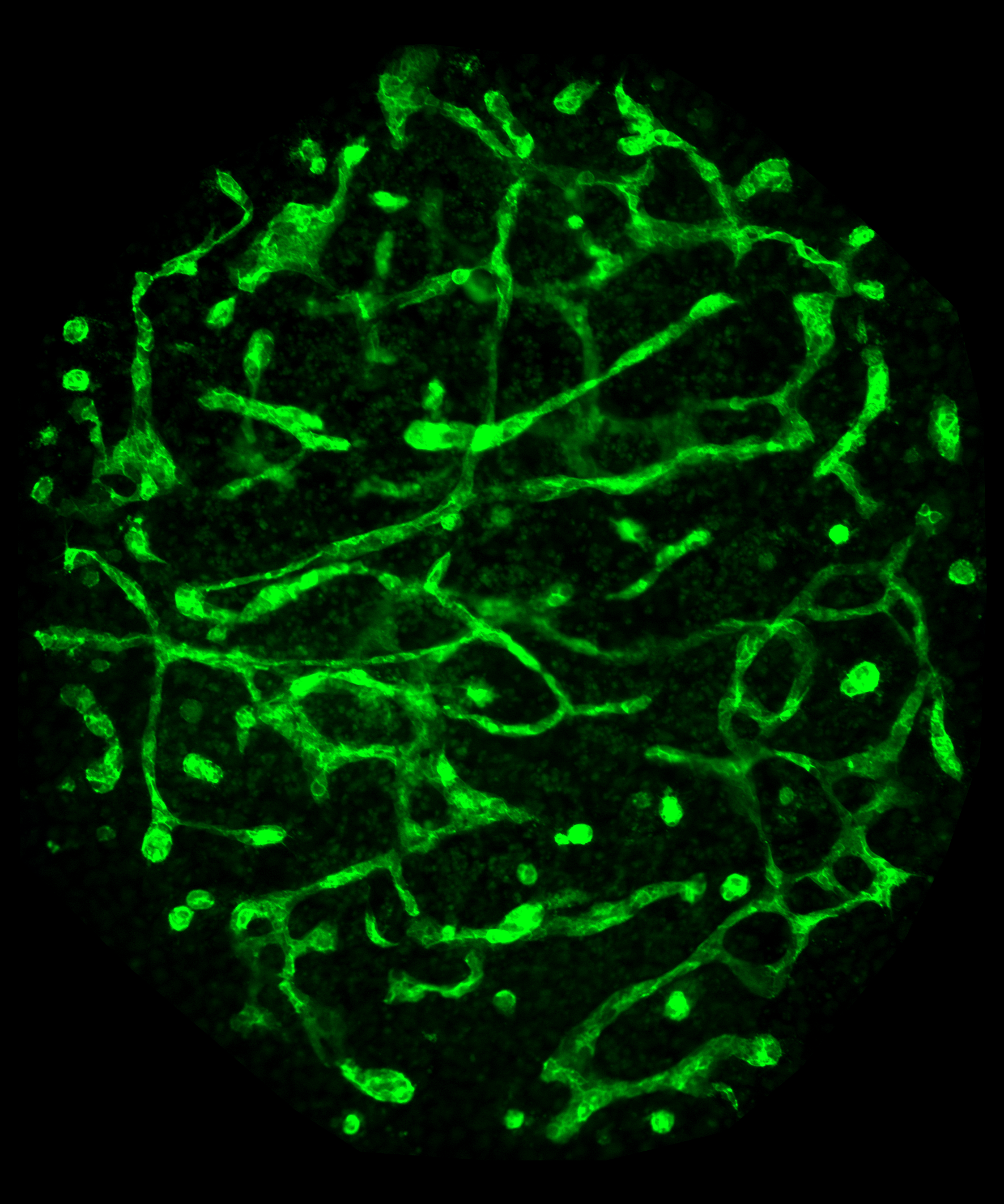

Supplement: Figure S20 — Microscopic image of a LY294002 treated allantois explant used for analysis shown in Figures 4 and 6 . (TIF) [file pone.0027385.s020.tif]

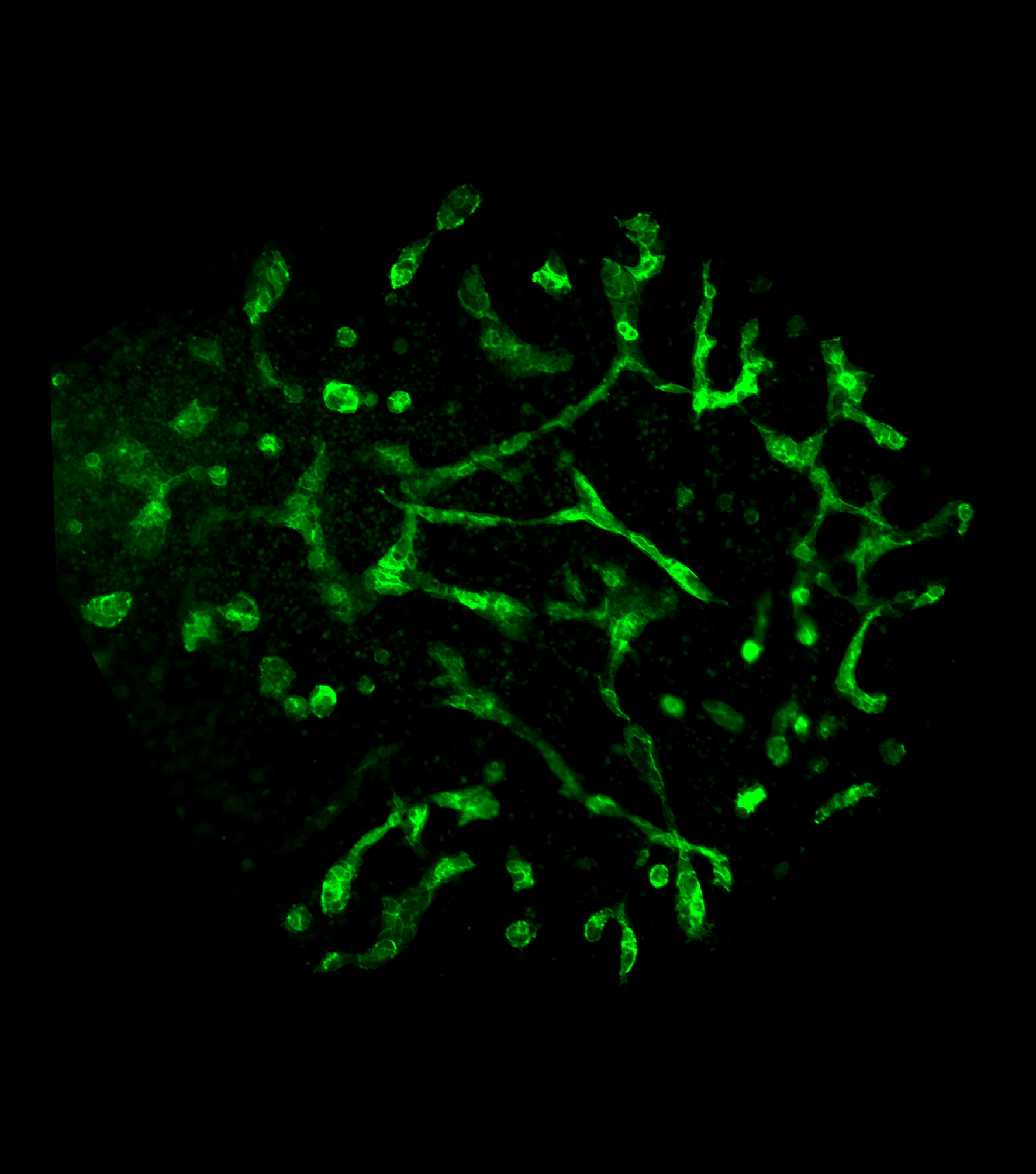

Supplement: Figure S21 — Microscopic image of a LY294002 treated allantois explant used for analysis shown in Figures 4 and 6 . (TIF) [file pone.0027385.s021.tif]

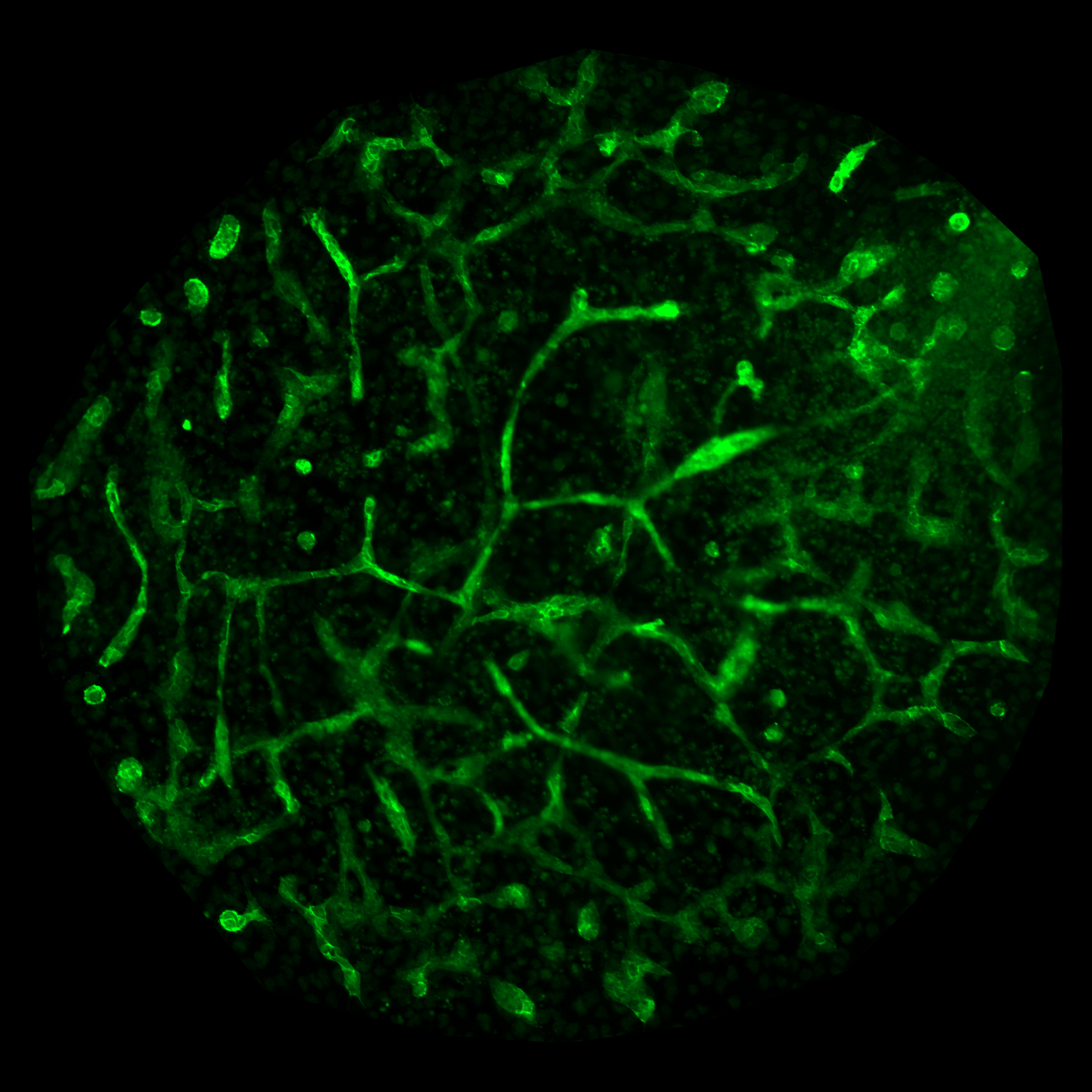

Supplement: Figure S22 — Microscopic image of a LY294002 treated allantois explant used for analysis shown in Figures 4 and 6 . (TIF) [file pone.0027385.s022.tif]

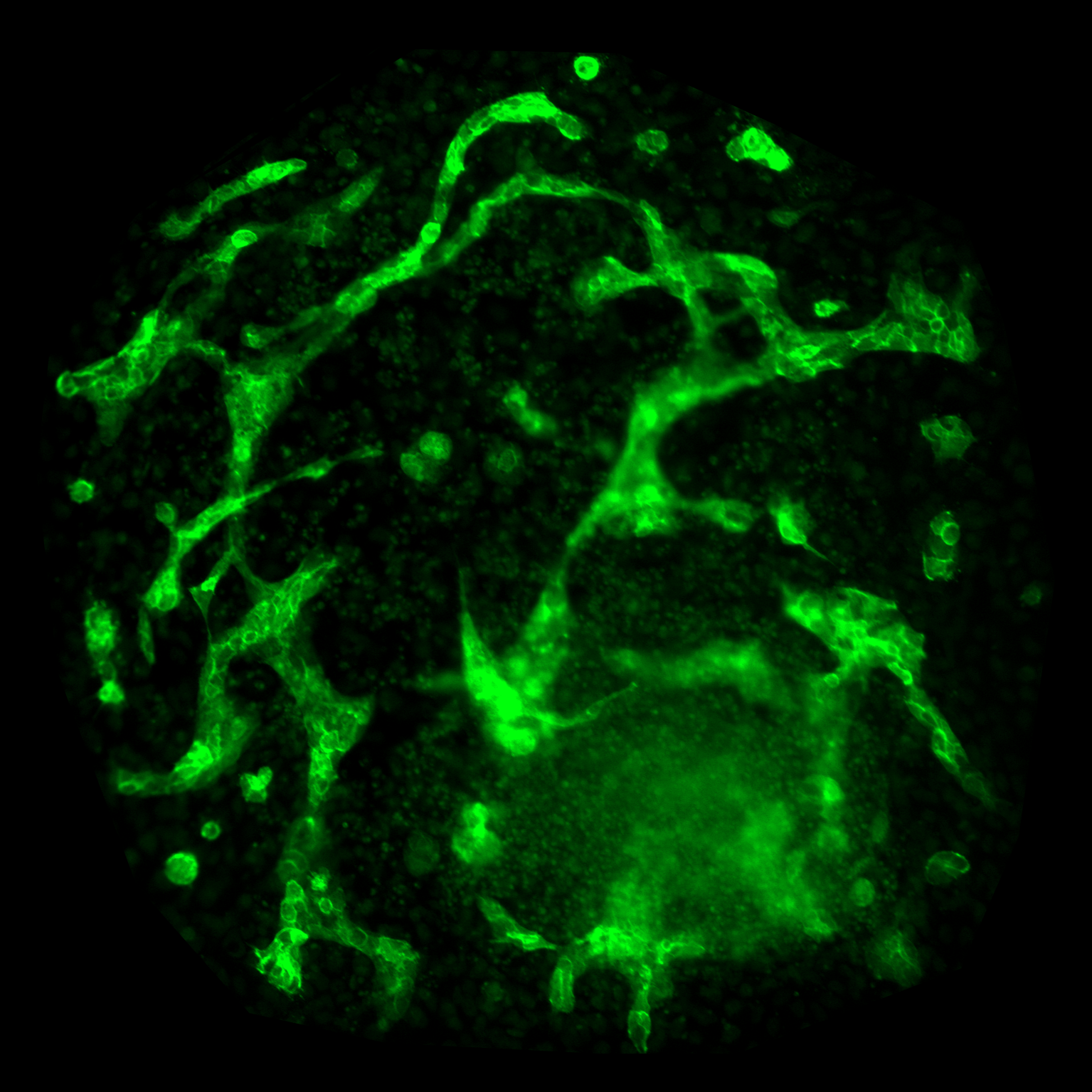

Supplement: Figure S23 — Microscopic image of a LY294002 treated allantois explant used for analysis shown in Figures 4 and 6 . (TIF) [file pone.0027385.s023.tif]

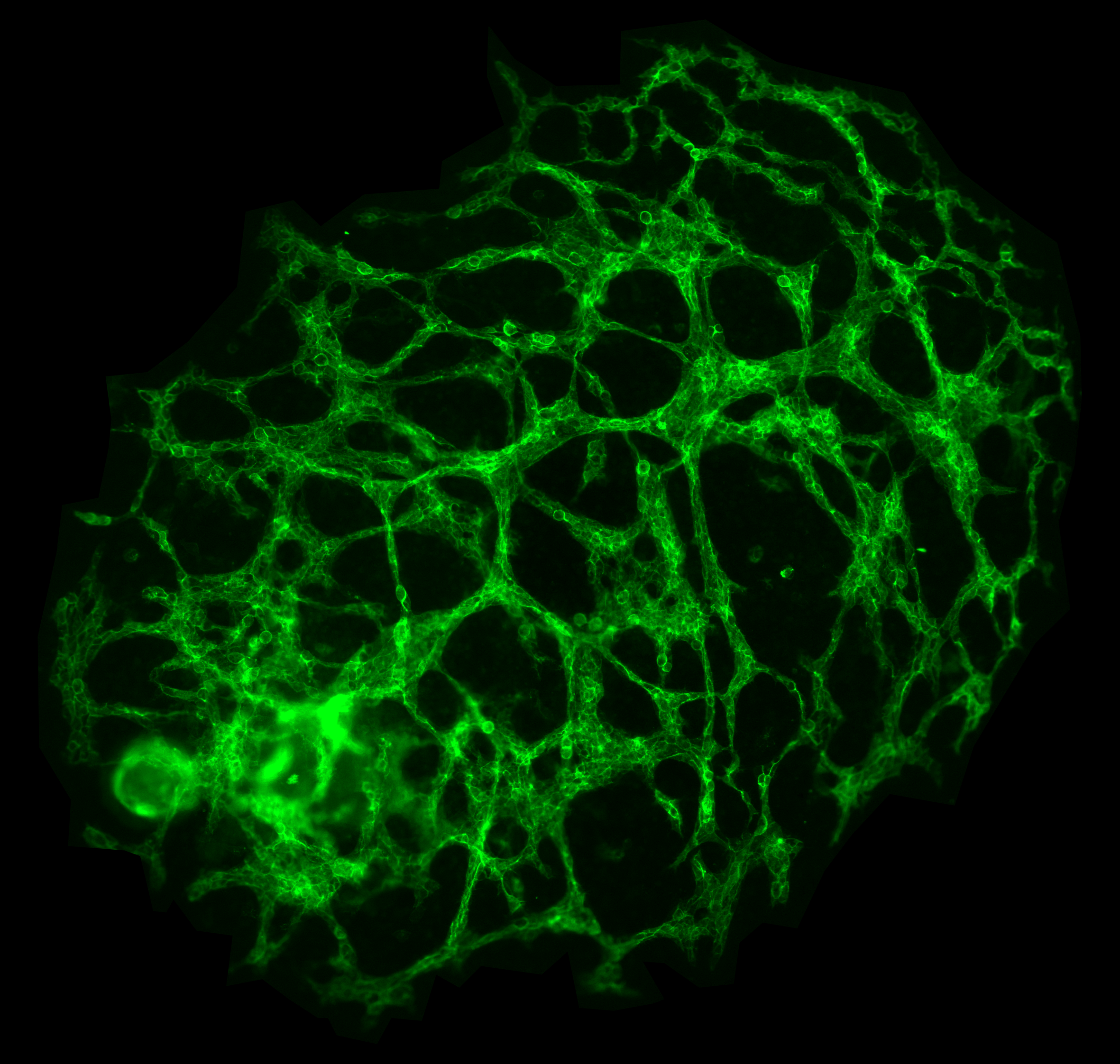

Supplement: Figure S24 — Microscopic image of a water treated control allantois explant used for analysis shown in Figure 5 . (TIF) [file pone.0027385.s024.tif]

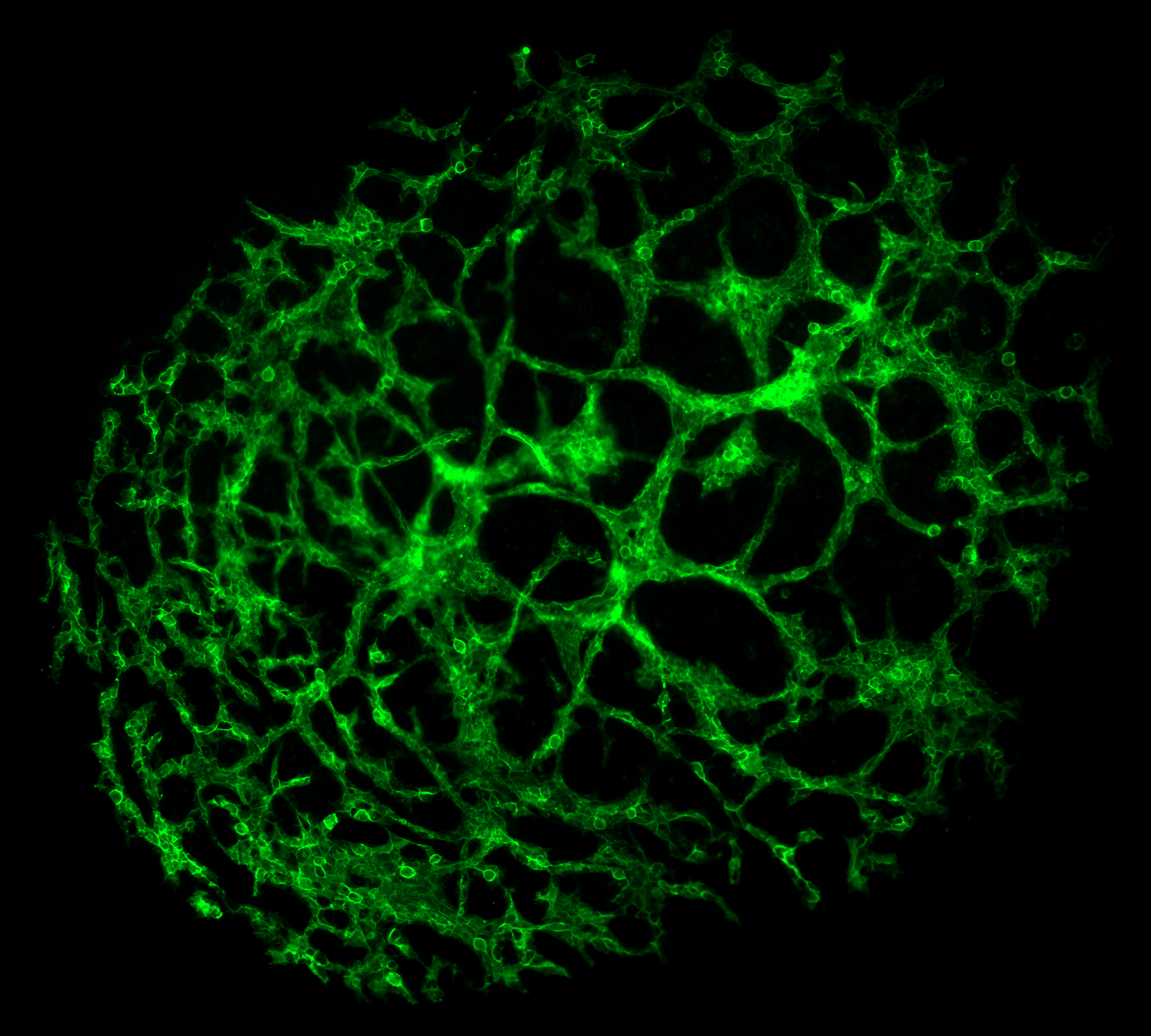

Supplement: Figure S25 — Microscopic image of a water treated control allantois explant used for analysis shown in Figure 5 . (TIF) [file pone.0027385.s025.tif]

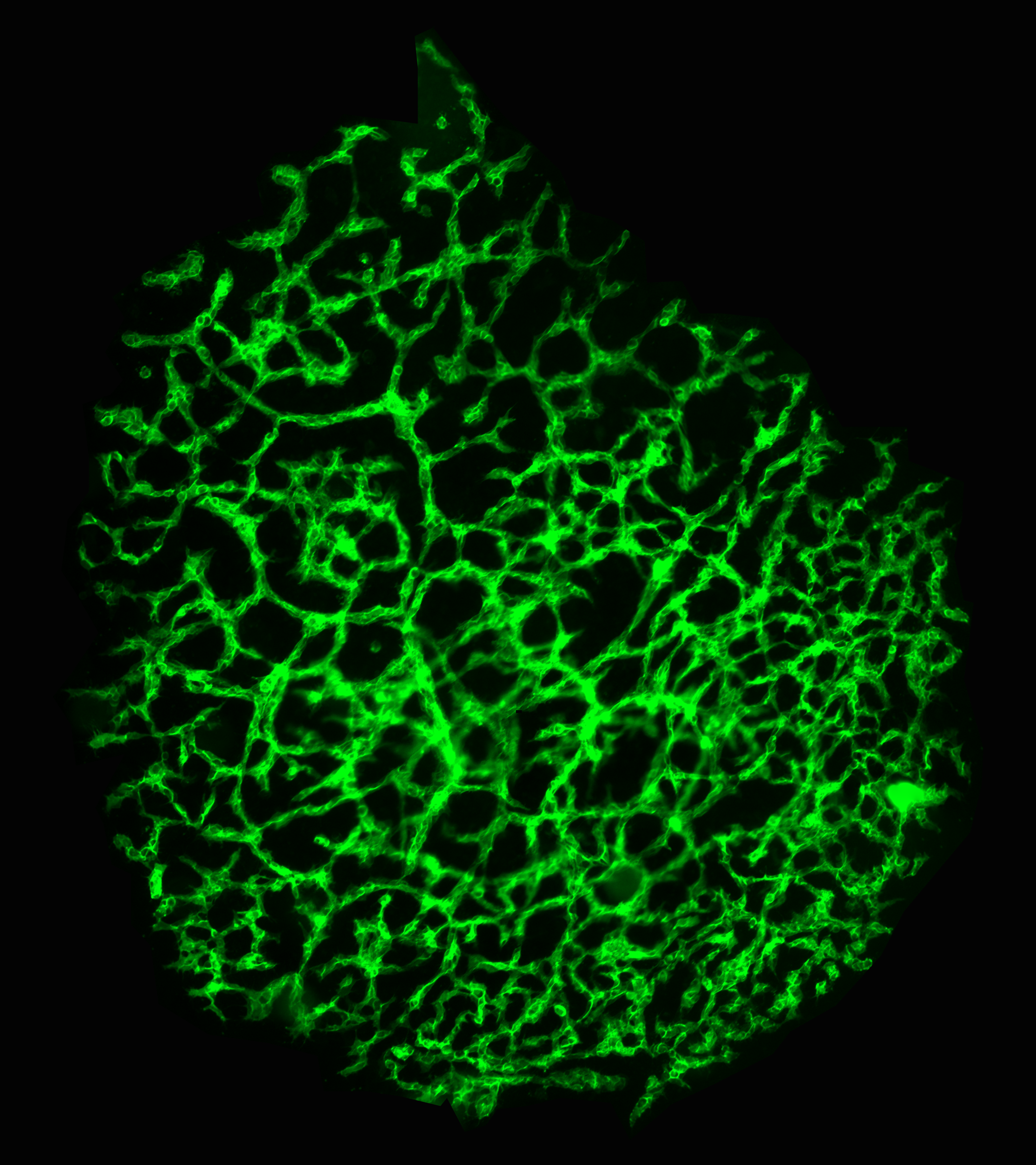

Supplement: Figure S26 — Microscopic image of a water treated control allantois explant used for analysis shown in Figure 5 . (TIF) [file pone.0027385.s026.tif]

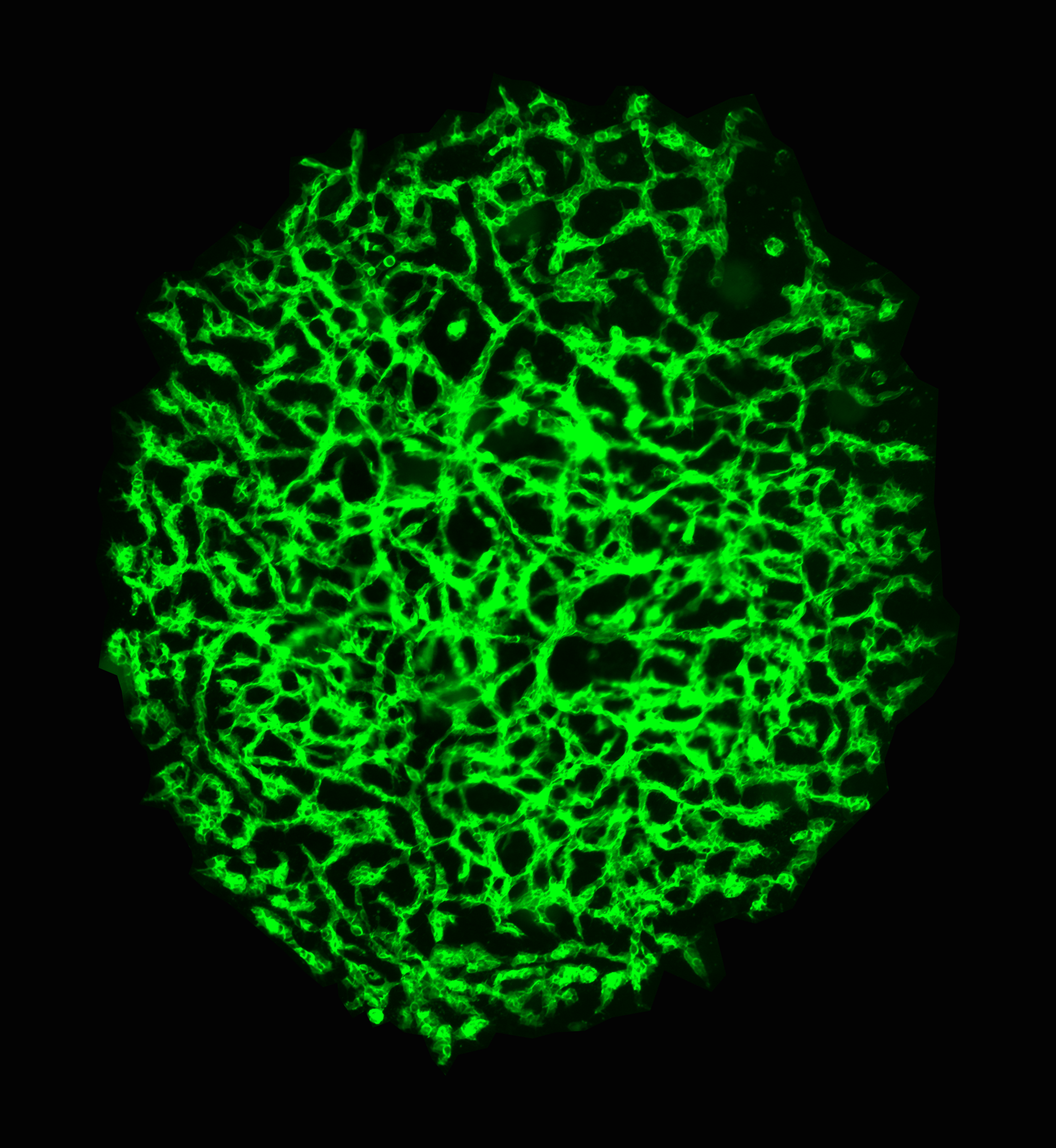

Supplement: Figure S27 — Microscopic image of a water treated control allantois explant used for analysis shown in Figure 5 . (TIF) [file pone.0027385.s027.tif]

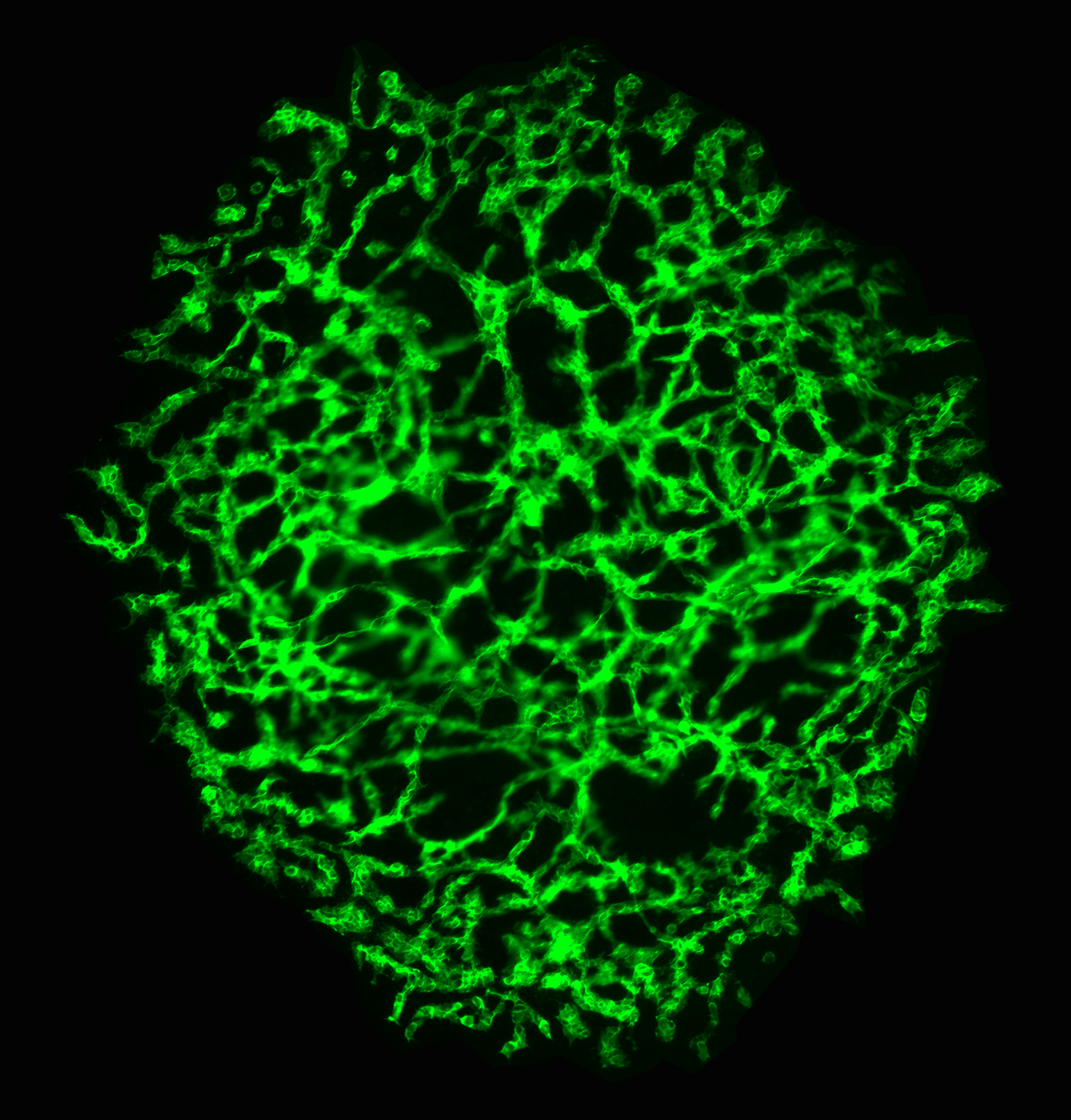

Supplement: Figure S28 — Microscopic image of a water treated control allantois explant used for analysis shown in Figure 5 . (TIF) [file pone.0027385.s028.tif]

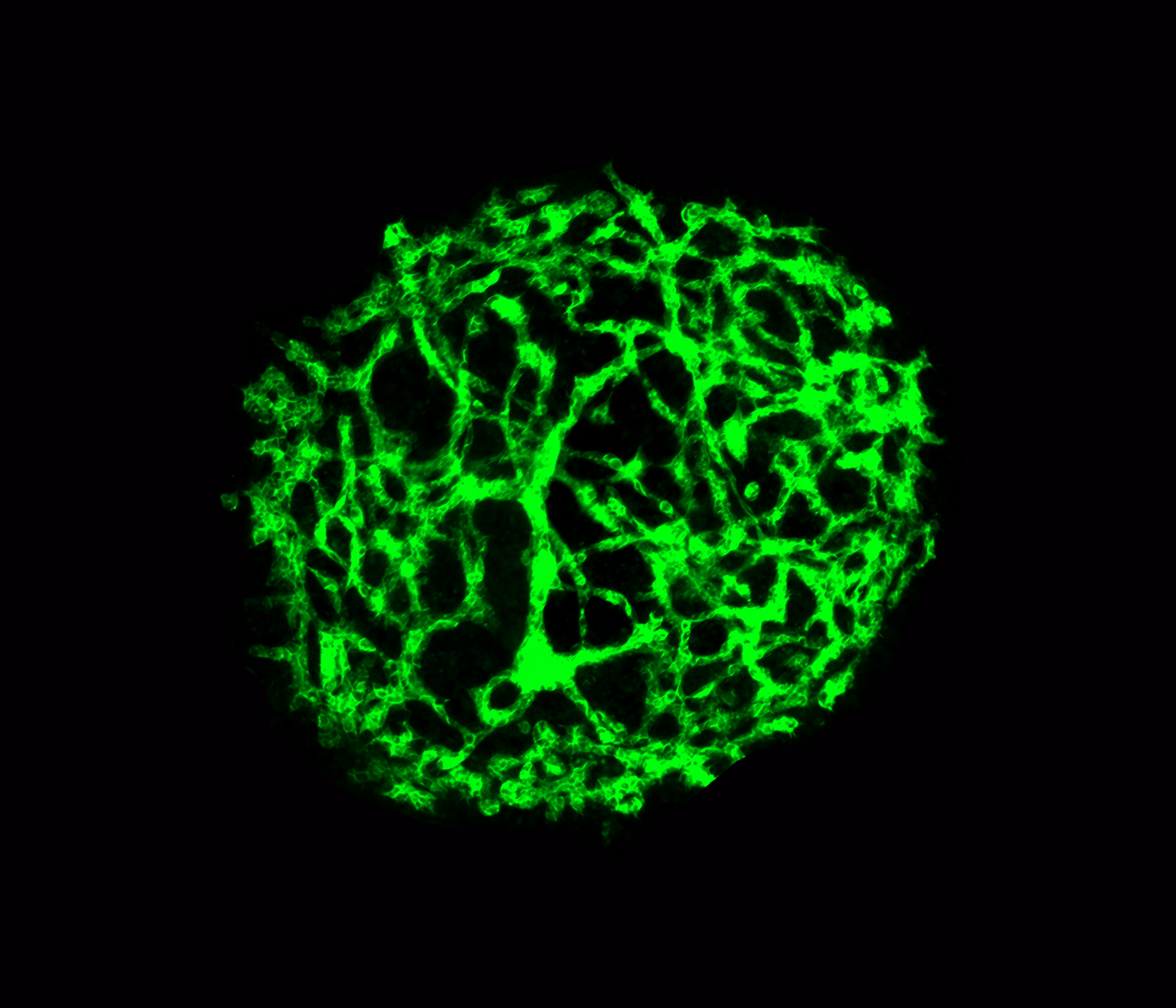

Supplement: Figure S29 — Microscopic image of a water treated control allantois explant used for analysis shown in Figure 5 . (TIF) [file pone.0027385.s029.tif]

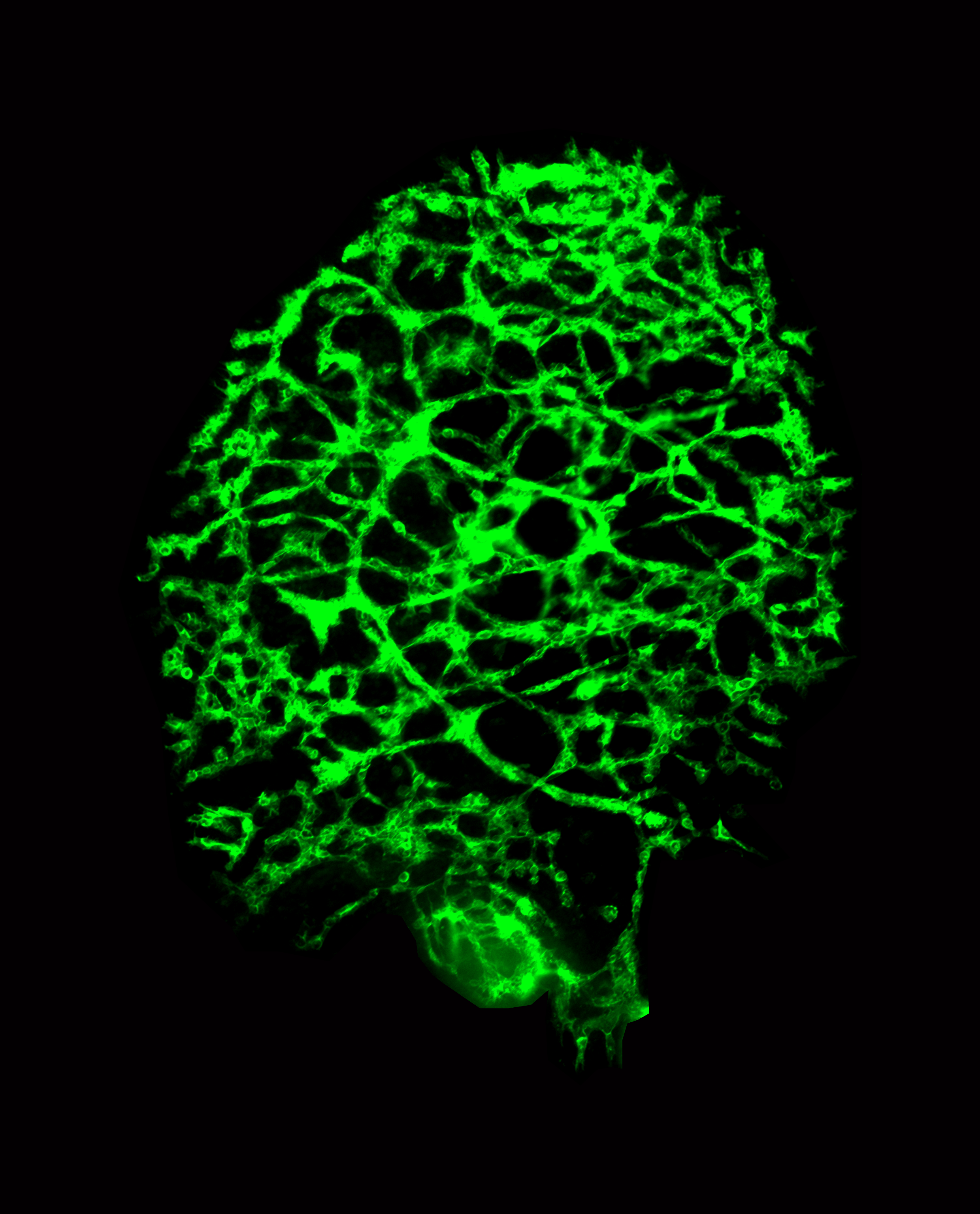

Supplement: Figure S30 — Microscopic image of a water treated control allantois explant used for analysis shown in Figure 5 . (TIF) [file pone.0027385.s030.tif]

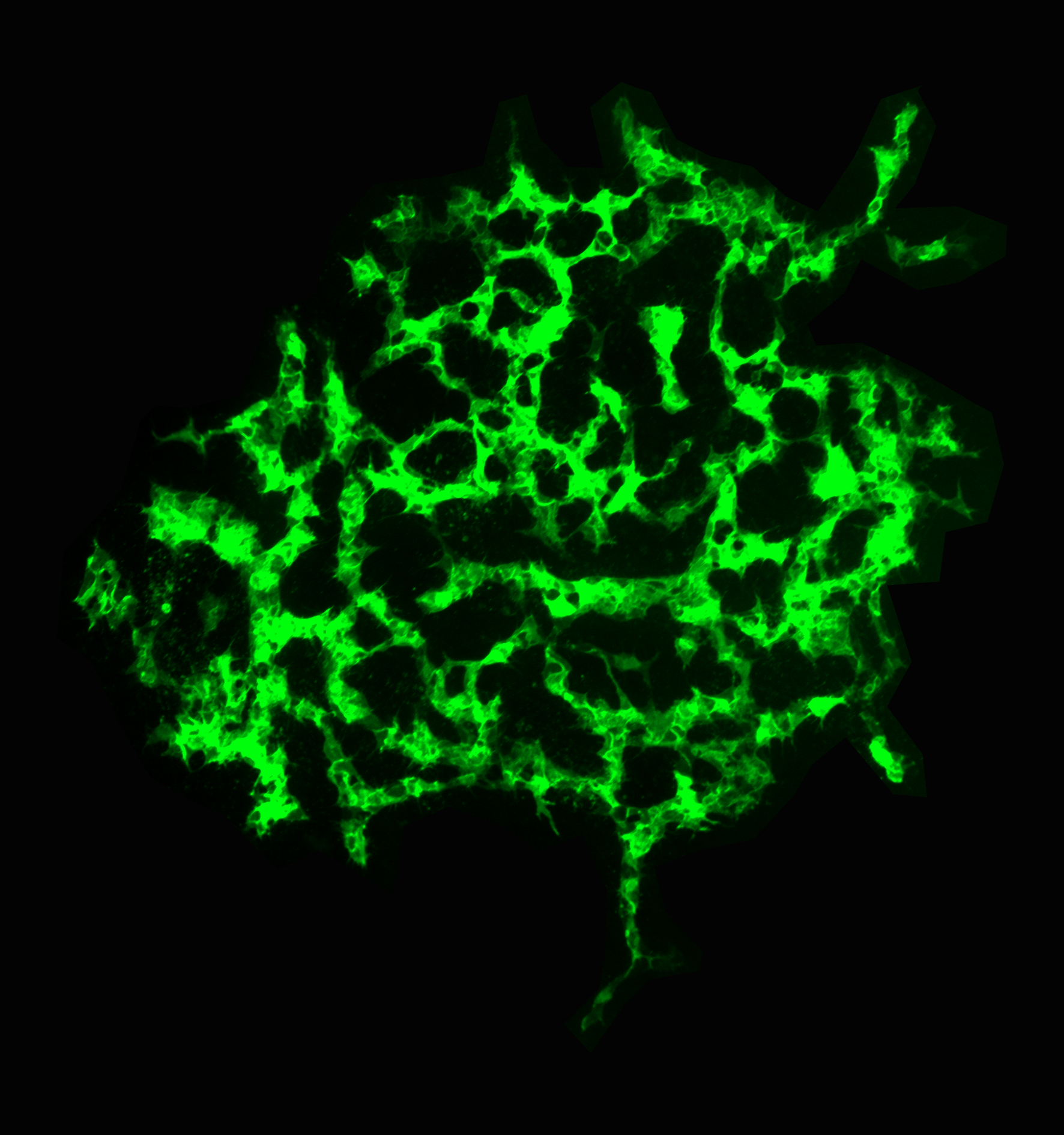

Supplement: Figure S31 — Microscopic image of a Y27632 treated allantois explant used for analysis shown in Figure 5 . (TIF) [file pone.0027385.s031.tif]

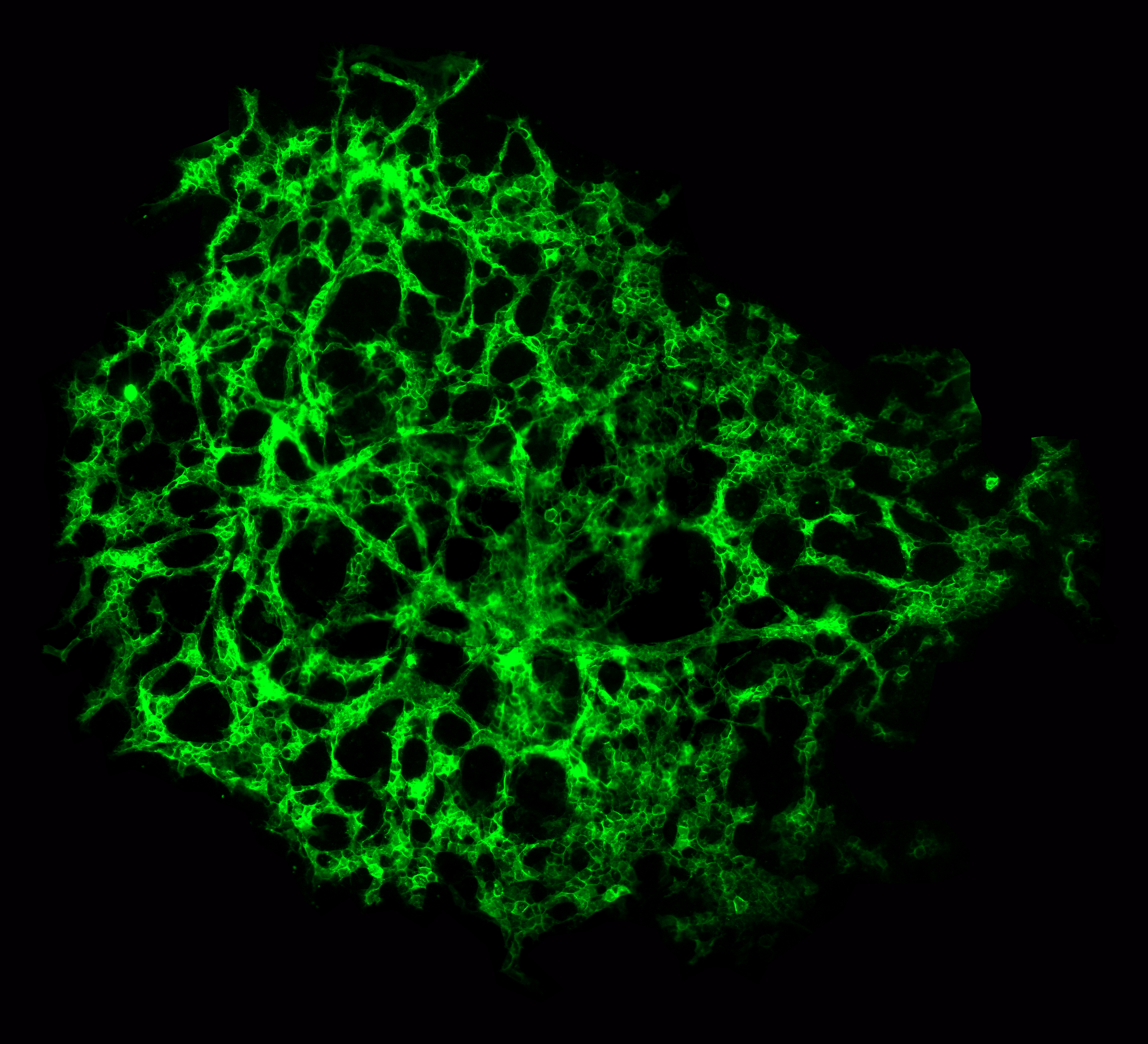

Supplement: Figure S32 — Microscopic image of a Y27632 treated allantois explant used for analysis shown in Figure 5 . (TIF) [file pone.0027385.s032.tif]

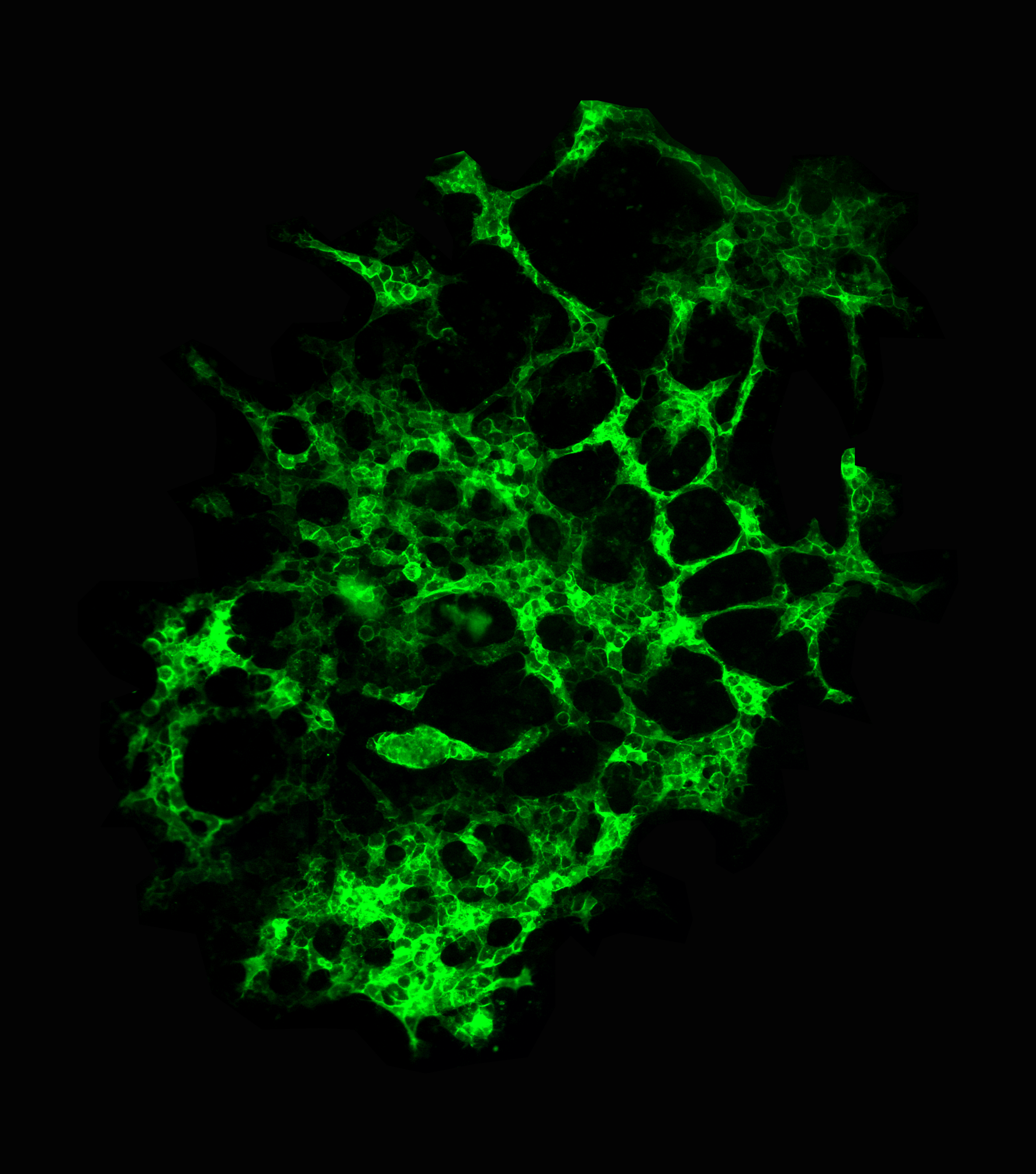

Supplement: Figure S33 — Microscopic image of a Y27632 treated allantois explant used for analysis shown in Figure 5 . (TIF) [file pone.0027385.s033.tif]

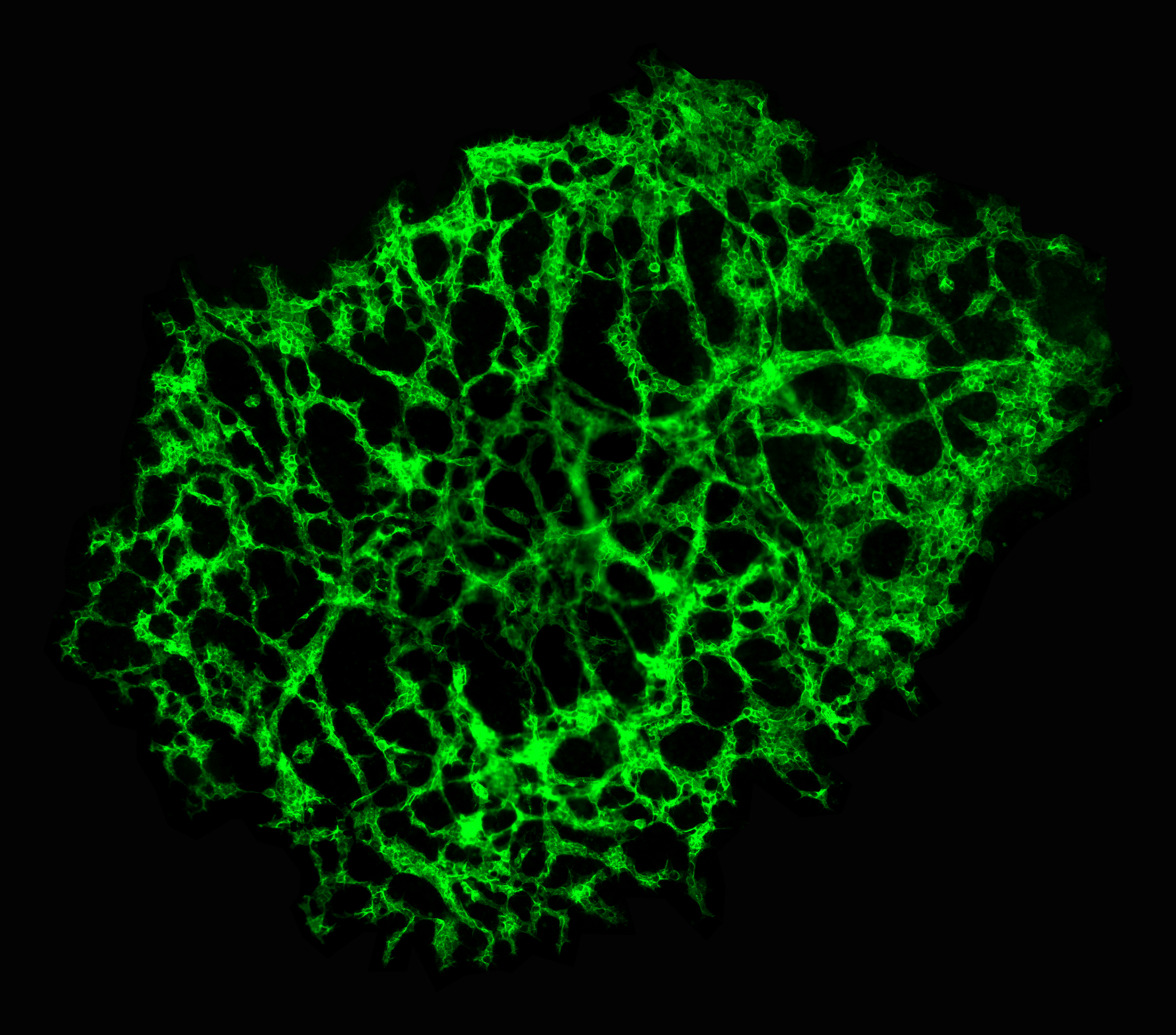

Supplement: Figure S34 — Microscopic image of a Y27632 treated allantois explant used for analysis shown in Figure 5 . (TIF) [file pone.0027385.s034.tif]

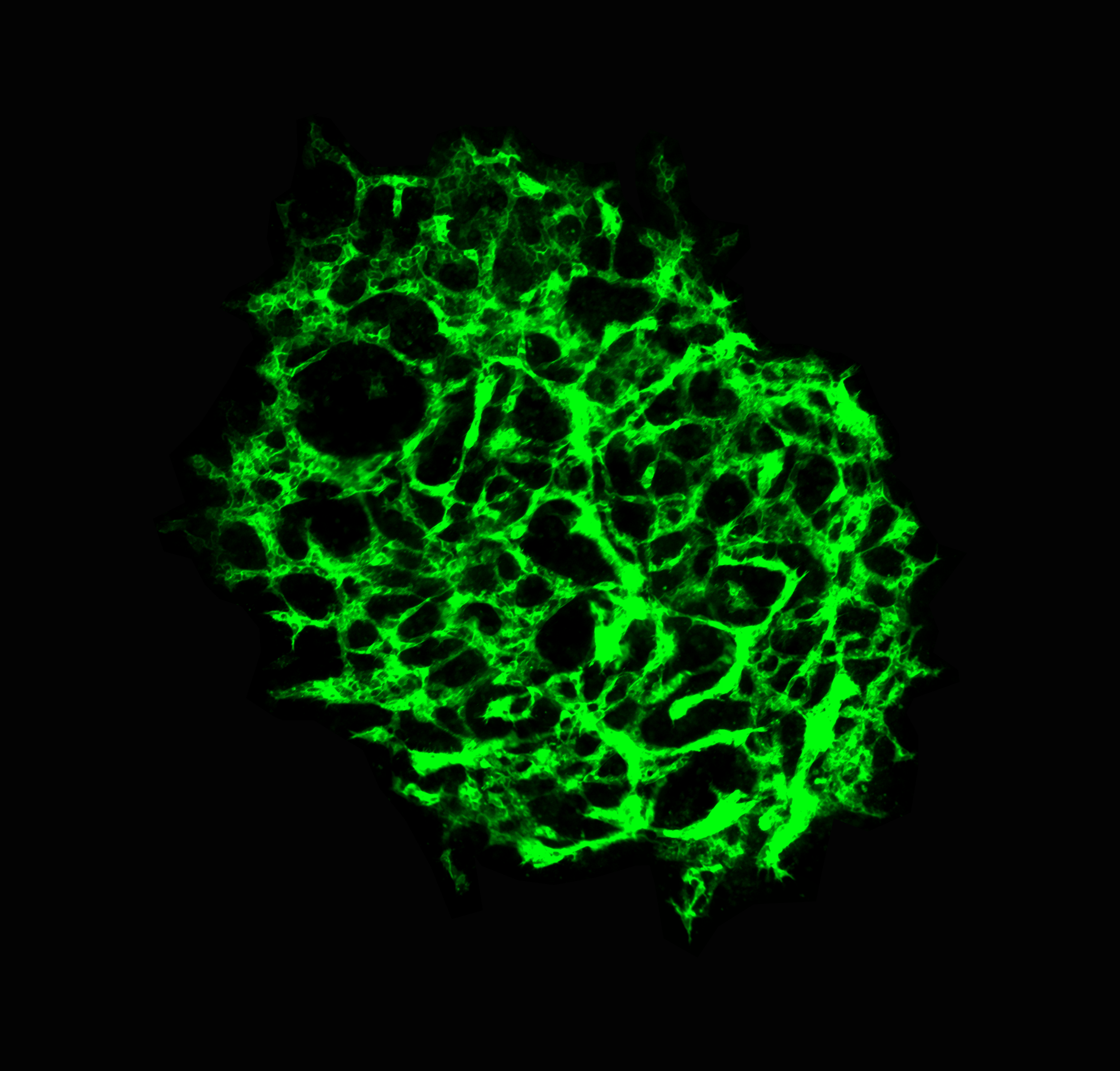

Supplement: Figure S35 — Microscopic image of a Y27632 treated allantois explant used for analysis shown in Figure 5 . (TIF) [file pone.0027385.s035.tif]

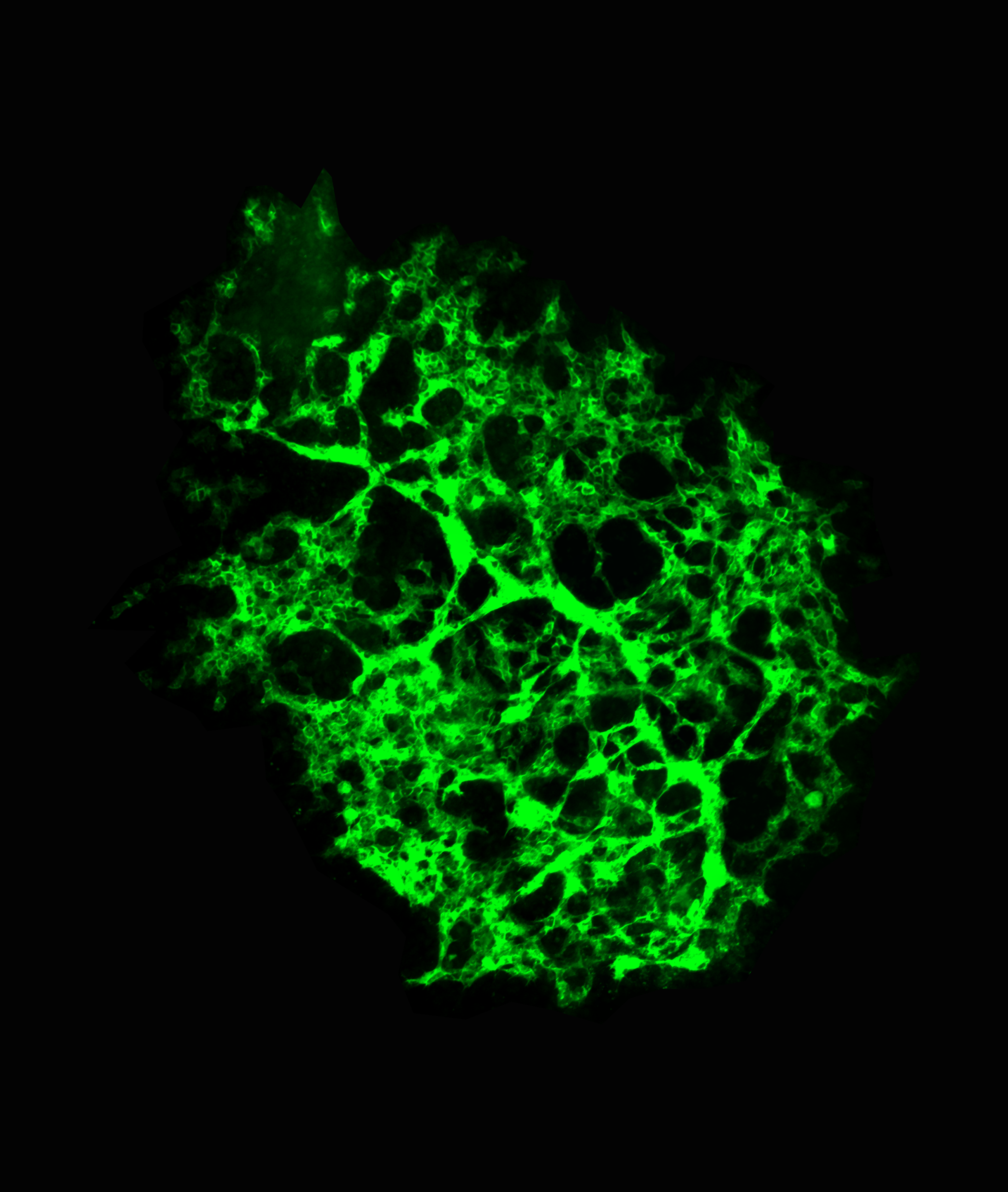

Supplement: Figure S36 — Microscopic image of a Y27632 treated allantois explant used for analysis shown in Figure 5 . (TIF) [file pone.0027385.s036.tif]

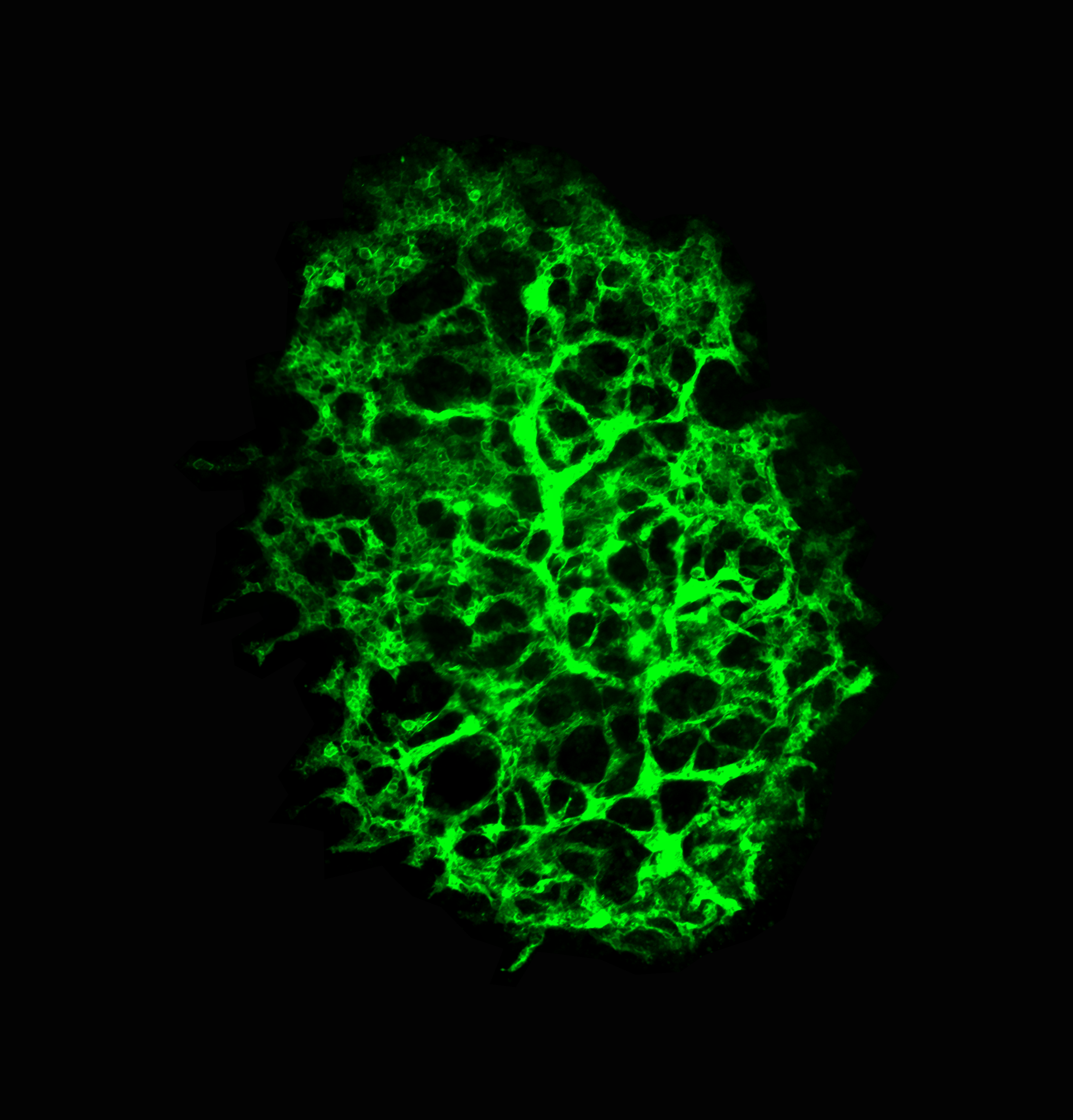

Supplement: Figure S37 — Microscopic image of a Y27632 treated allantois explant used for analysis shown in Figure 5 . (TIF) [file pone.0027385.s037.tif]

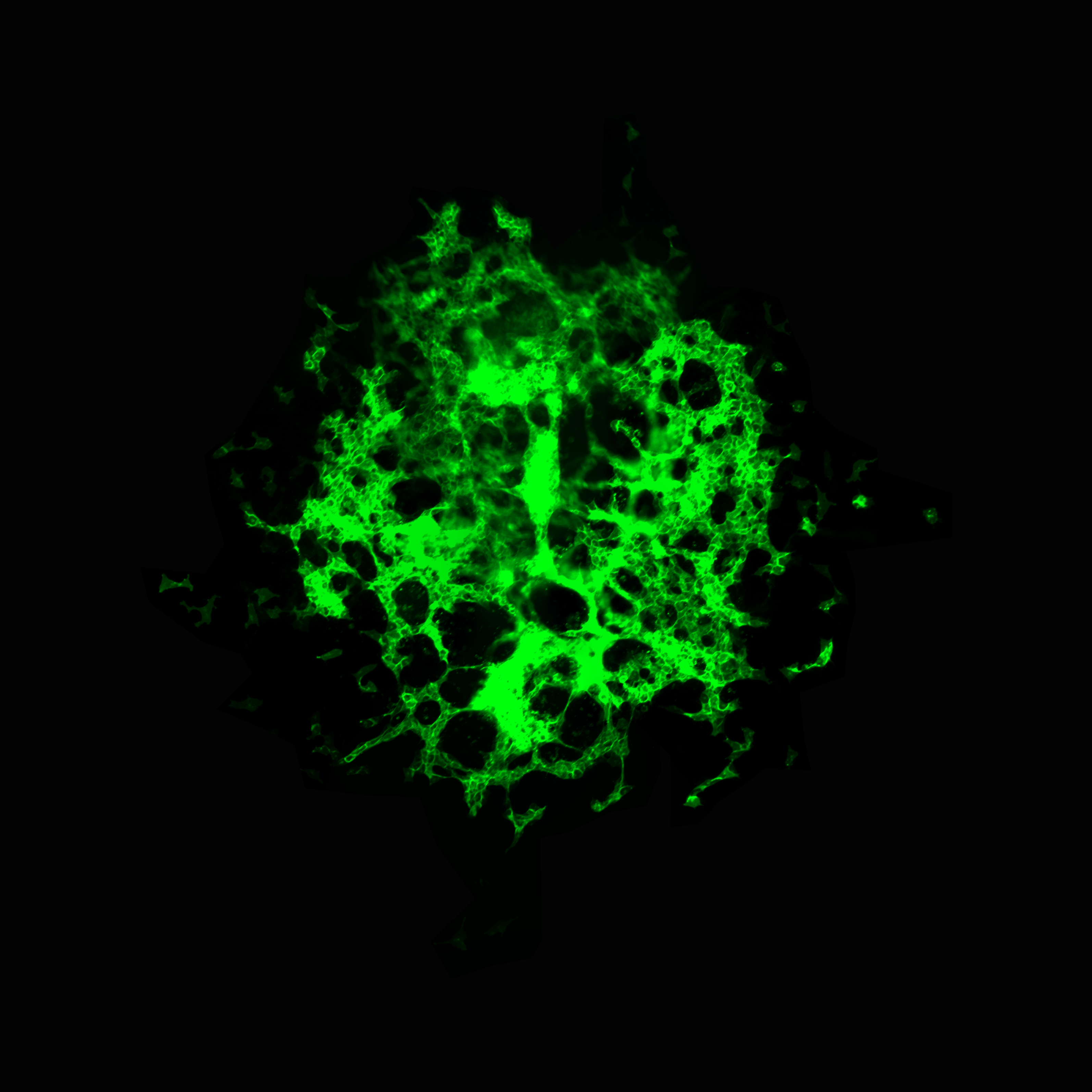

Supplement: Figure S38 — Microscopic image of a Y27632 treated allantois explant used for analysis shown in Figure 5 . (TIF) [file pone.0027385.s038.tif]

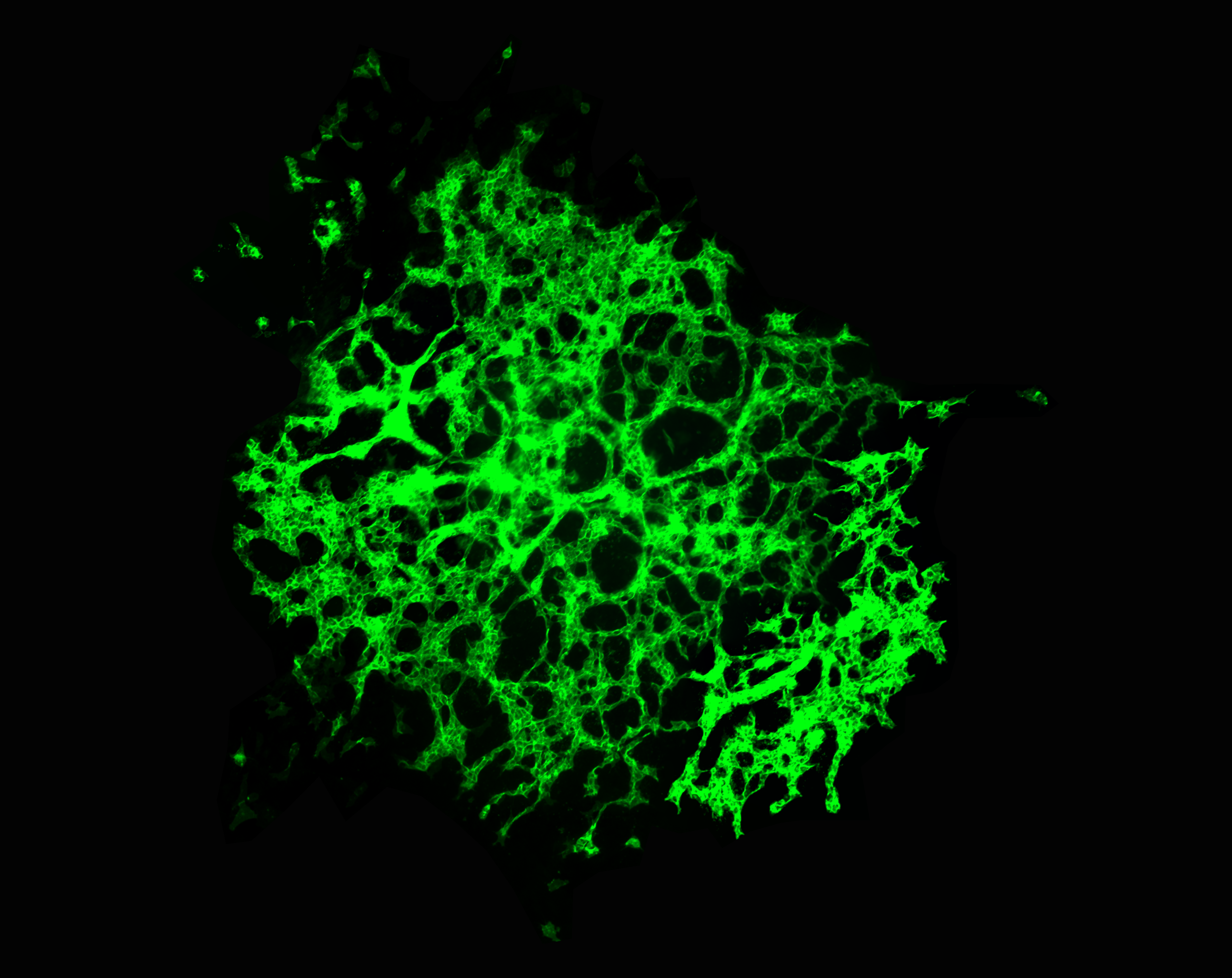

Supplement: Figure S39 — Microscopic image of a Y27632 treated allantois explant used for analysis shown in Figure 5 . (TIF) [file pone.0027385.s039.tif]

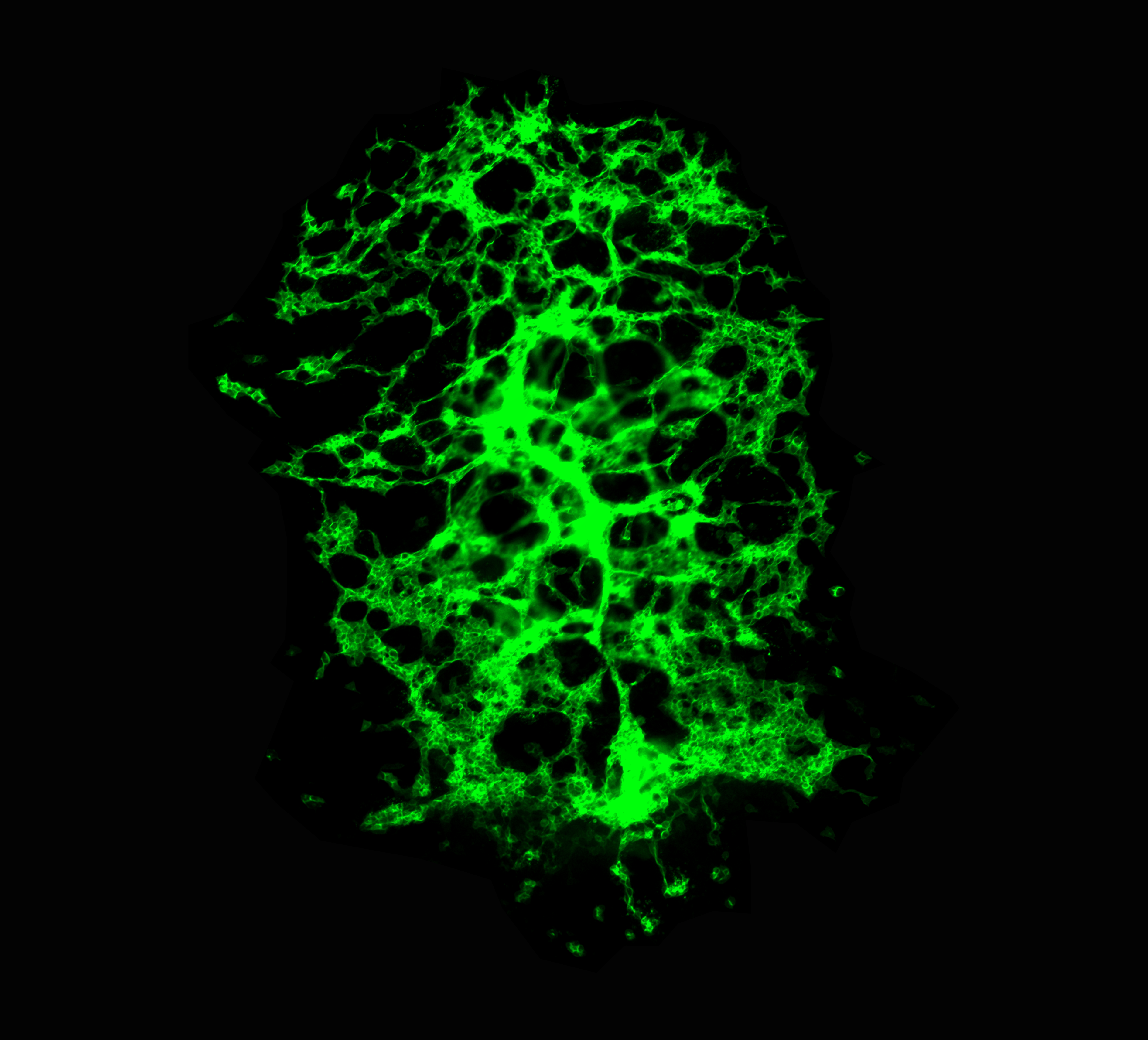

Supplement: Figure S40 — Microscopic image of a Y27632 treated allantois explant used for analysis shown in Figure 5 . (TIF) [file pone.0027385.s040.tif]
